# Phenotypic and pharmacogenetic evaluation of patients with thiazide-induced hyponatremia

**DOI:** 10.1172/JCI89812

**Published:** 2017-08-07

**Authors:** James S. Ware, Louise V. Wain, Sarath K. Channavajjhala, Victoria E. Jackson, Elizabeth Edwards, Run Lu, Keith Siew, Wenjing Jia, Nick Shrine, Sue Kinnear, Mahli Jalland, Amanda P. Henry, Jenny Clayton, Kevin M. O’Shaughnessy, Martin D. Tobin, Victor L. Schuster, Stuart Cook, Ian P. Hall, Mark Glover

**Affiliations:** 1 NIHR Biomedical Research Unit in Cardiovascular Disease at Royal Brompton & Harefield, NHS Foundation Trust and Imperial College London, London, United Kingdom.; 2 National Heart and Lung Institute, Imperial College London, London, United Kingdom.; 3 Genetic Epidemiology Group, Department of Health Sciences, University of Leicester, Leicester, United Kingdom.; 4 Division of Therapeutics and Molecular Medicine, University of Nottingham, Nottingham, United Kingdom.; 5 NIHR Nottingham Biomedical Research Centre, Nottingham, United Kingdom.; 6 Albert Einstein College of Medicine, Yeshiva University, New York, New York, USA.; 7 Clinical Pharmacology Unit, University of Cambridge, Cambridge, United Kingdom.; 8 Department of Diabetes and Endocrinology, Nottingham University Hospitals NHS Trust, Nottingham, United Kingdom.; 9 Duke-National University of Singapore, Singapore.; 10 National Heart Centre Singapore, Singapore.

**Keywords:** Nephrology, Therapeutics, Hypertension

## Abstract

Thiazide diuretics are among the most widely used treatments for hypertension, but thiazide-induced hyponatremia (TIH), a clinically significant adverse effect, is poorly understood. Here, we have studied the phenotypic and genetic characteristics of patients hospitalized with TIH. In a cohort of 109 TIH patients, those with severe TIH displayed an extended phenotype of intravascular volume expansion, increased free water reabsorption, urinary prostaglandin E_2_ excretion, and reduced excretion of serum chloride, magnesium, zinc, and antidiuretic hormone. GWAS in a separate cohort of 48 TIH patients and 2,922 controls from the 1958 British birth cohort identified an additional 14 regions associated with TIH. We identified a suggestive association with a variant in *SLCO2A1*, which encodes a prostaglandin transporter in the distal nephron. Resequencing of *SLCO2A1* revealed a nonsynonymous variant, rs34550074 (p.A396T), and association with this SNP was replicated in a second cohort of TIH cases. TIH patients with the p.A396T variant demonstrated increased urinary excretion of prostaglandin E_2_ and metabolites. Moreover, the SLCO2A1 phospho-mimic p.A396E showed loss of transporter function in vitro. These findings indicate that the phenotype of TIH involves a more extensive metabolic derangement than previously recognized. We propose one mechanism underlying TIH development in a subgroup of patients in which SLCO2A1 regulation is altered.

## Introduction

Cardiovascular disease is the leading cause of mortality worldwide (1, 2) and hypertension one of its most important modifiable causes. Thiazide diuretics inhibit the thiazide-sensitive sodium chloride cotransporter NCC in the distal convoluted tubule (DCT) of the kidney (3) and are among the most widely used class of medicines in the management of hypertension (4).

However, some patients given thiazides develop thiazide-induced hyponatremia (TIH) (5). Severe TIH (serum sodium <125 mM) causes debilitating symptoms (5) and is the most common form of drug-induced hyponatremia requiring hospital admission (6). The mechanism of TIH is poorly understood. Serum sodium concentration in the thiazide-treated general population is virtually unchanged by thiazide therapy (7), implying that TIH occurs in a susceptible subgroup, but that this subgroup cannot be prospectively identified, and so TIH is largely unpredictable at the point of thiazide initiation. Pharmacogenetic predisposition to a range of adverse drug effects (8–14) raises the possibility that TIH might also have genetic causation. This hypothesis is supported by the highly reproducible nature of TIH even on single-dose rechallenge in individuals for whom environmental factors are controlled (15–17). We therefore set out to study the phenotypic and genetic characteristics of 2 cohorts of patients admitted to the hospital with severe symptomatic TIH in the United Kingdom.

## Results

### Characteristics of cohort 1 and cohort 2 TIH cases and controls

**Figure 1:**
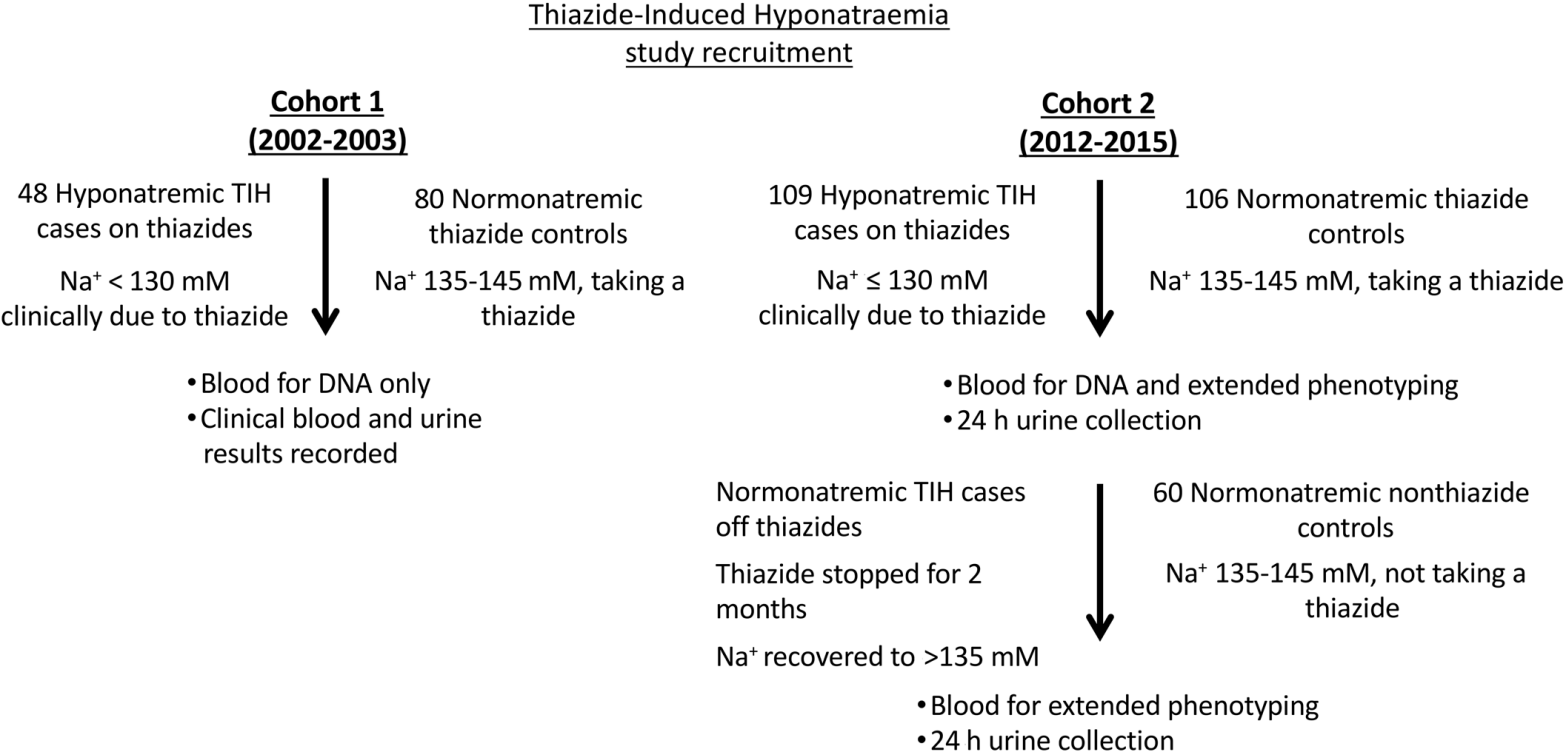
An overview of study recruitment of TIH cases and controls in cohort 1 and cohort 2. Cohort 1 hyponatremic TIH cases on thiazides with serum sodium levels of less than 130 mM were recruited in 2002 and 2003 together with matched cohort 1 normonatremic thiazide controls. Blood was taken for DNA and clinical details and investigations recorded. Cohort 2 hyponatremic TIH cases on thiazides with serum sodium levels of less than 130 mM were recruited from 2012 to 2015. Blood was taken together with 24-hour urine collection. DNA was extracted from the blood and extensive electrolyte and hormonal phenotyping undertaken. TIH cases were reviewed and phenotyping blood and urine samples repeated after 2 months off thiazide (termed cohort 2 normonatremic TIH cases off thiazides). Two matched control groups were recruited in cohort 2; the first were normonatremic and took thiazides (termed cohort 2 normonatremic thiazide controls), and the second were normonatremic but did not take thiazides (termed cohort 2 normonatremic nonthiazide controls).

Two cohorts of patients of mixed European descent who were hospitalized with symptomatic TIH were recruited (Figure 1, Methods, and Supplemental Methods; supplemental material available online with this article; https://doi.org/10.1172/JCI89812DS1). The characteristics of the TIH cases and controls from cohorts 1 and 2 are presented in Table 1. In both cohorts, hyponatremic TIH cases on thiazides were typically aged over 70 years, with a predominance of females (Table 1).

### Phenotypic differences between hyponatremic TIH cases on thiazides and normonatremic thiazide controls

Cohort 1 hyponatremic TIH cases on thiazides had lower serum potassium compared with cohort 1 normonatremic thiazide controls (Supplemental Table 2). In cohort 2, hyponatremic TIH cases on thiazides also demonstrated significantly (*P* < 10^-5^) lower serum potassium on treatment compared with off treatment. Cohort 2 hyponatremic TIH cases on thiazides also had lower blood pressure, lower blood glucose, and a lower serum concentration of chloride, magnesium, calcium, zinc, and vitamin D relative to cohort 2 normonatremic thiazide controls.

Twenty-four hour urine volume and osmolarity were significantly lower in hyponatremic TIH cases on thiazides compared with normonatremic thiazide controls (Supplemental Table 3). Fractional urinary excretion of potassium and phosphate was reduced and that of zinc was increased (Supplemental Table 4). Twenty-four hour urinary urea excretion, an important determinant of free water clearance, was also significantly lower in TIH cases both on and off thiazide, compared with relevant controls (Supplemental Table 3).

### Evaluating the baseline physiology of TIH cases after thiazide withdrawal

Serum abnormalities resolved following thiazide cessation with the exception of levels of chloride and zinc (Supplemental Table 2). Although hypochloridemia and hypozincemia improved following thiazide cessation, cohort 2 normonatremic case remained hypochloridemic and hypozincemic 2 months after stopping thiazide therapy (Supplemental Table 2).

### TIH cases display an exaggerated increase in free water reabsorption

Cohort 2 TIH cases reabsorbed 48% more free water when on thiazides compared with when off thiazides, which is in marked contrast with cohort 2 controls, who showed only a 9% increase in free water reabsorption while on thiazide (Supplemental Table 3). This suggests that TIH cases display an exaggerated increase in free water reabsorption in response to thiazide exposure. All groups in cohort 2 were in a state of net free water reabsorption (Supplemental Table 3). Although solute-free water reabsorption was lower in cohort 2 hyponatremic TIH cases on thiazide than in cohort 2 normonatremic thiazide controls, continued water reabsorption and production of a concentrated urine by hyponatremic TIH cases on thiazides is clearly inappropriate in the context of profound hyponatremia and intravascular volume expansion (as assessed by increased fractional urate clearance).

### Increased fractional uric acid clearance in TIH suggests volume expansion

**Figure 2:**
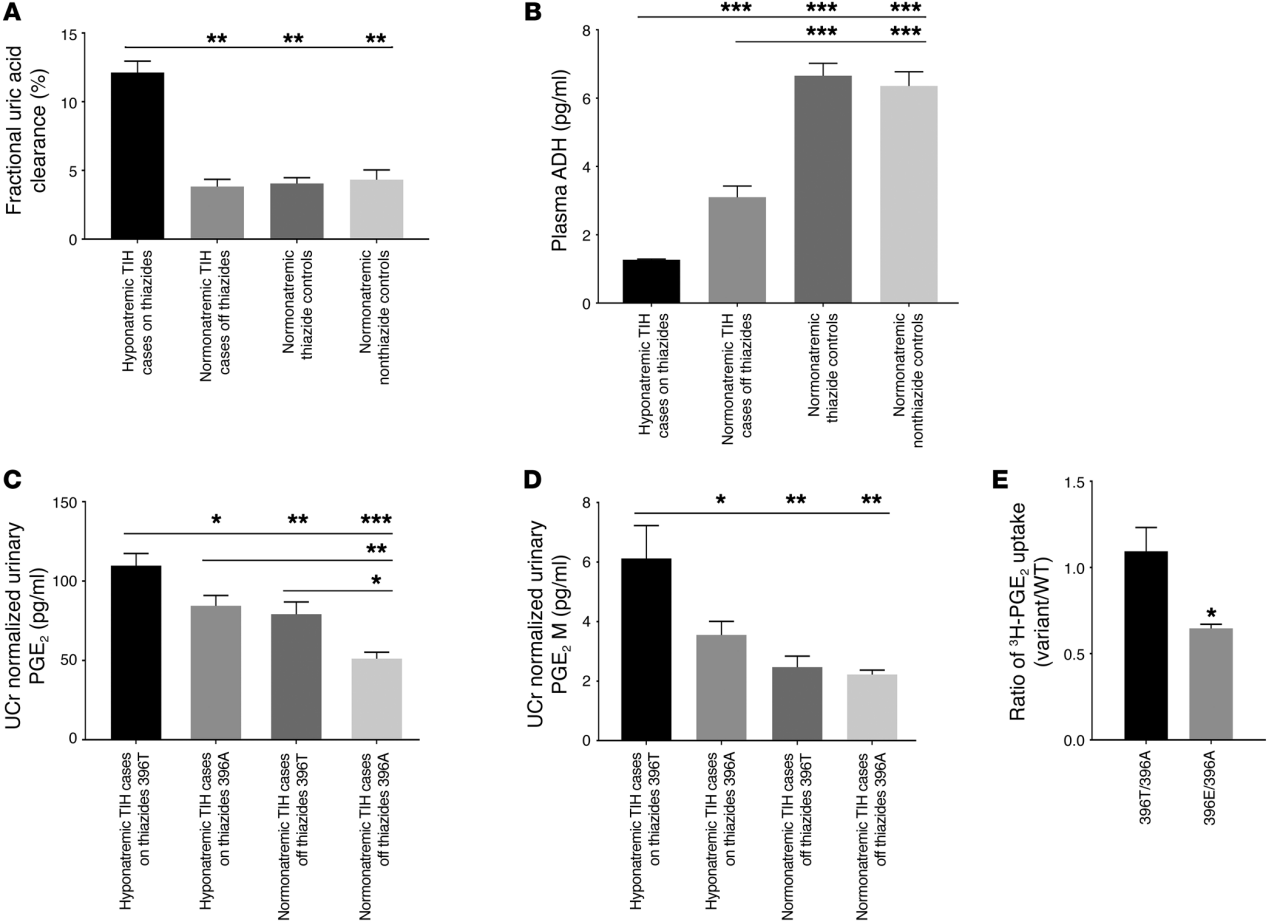
Phenotypic characteristics of TIH cases and controls and in vitro activity of SLCO2A1 (PGT) site mutants. (**A**) Fractional renal uric acid clearance in patients in cohort 2 TIH cases and controls. Fractional uric acid clearance is increased in hyponatremic TIH cases on thiazides compared with controls, suggesting volume expansion. *n* = 20 in each group. (**B**) Plasma ADH concentration in cohort 2 TIH cases and controls. ADH is lower in hyponatremic TIH cases on thiazides compared with controls. *n* = 20 in each group. (**C** and **D**) Urinary PGE_2_ and PGE_2_M concentration in cohort 2 TIH cases by SLCO2A1 p.396 allele. p.396T, *n* = 22; p.396A, *n* = 25, (**E**) Rate of ^3^H-PGE_2_ uptake (fmol PGE_2_/mg protein/10 min) by human SLCO2A1 expressed transiently in HEK293 cells. Data are presented as ratio of ^3^H-PGE_2_ uptake (396T/396A, left, *n* = 5 paired experiments, 396T = 37.8 ± 8.1, 396A = 33.5 ± 4.0, *P* = 0.44; r396E/396A, right, *n* = 3 paired experiments, 396A = 35.7 ± 6.2, 396E = 23.1 ± 4.6, *P* = 0.02). Data are represented as mean ± SEM. *P < 0.05; **P < 0.01; ***P < 0.001. Comparisons in **A**–**D** were determined by 1-way ANOVA with Bonferroni’s correction. Comparison in **E** was determined by 2-tailed Student’s *t* test. Ucr, urinary creatinine; Ctrl, control.

Increased fractional renal excretion of uric acid is observed in the syndrome of inappropriate antidiuretic hormone secretion (SIADH) and is caused by arterial blood volume expansion (18, 19). Mean serum and urinary uric acid concentration in cohort 2 hyponatremic TIH cases on thiazides was significantly lower than in cohort 2 normonatremic thiazide controls (Supplemental Figure 1). Fractional uric acid clearance in cohort 2 hyponatremic TIH cases on thiazides was significantly increased compared with both normonatremic thiazide and nonthiazide control groups and recovered to normal following thiazide cessation (Figure 2A).

### Plasma antidiuretic hormone concentration is suppressed during TIH

Mean plasma antidiuretic hormone (ADH) concentration in cohort 2 hyponatremic TIH cases on thiazides was significantly lower than in cohort 2 normonatremic thiazide controls and normonatremic TIH cases off thiazides (Figure 2B). Although plasma ADH concentration increased in TIH cases after thiazide cessation and resolution of hyponatremia, ADH remained lower in normonatremic TIH cases off thiazide than in normonatremic nonthiazide controls.

### Results of genetic studies

#### GWAS.

We undertook a GWAS using the cohort 1 cases and controls from the 1958 British birth cohort. Given the limited number of cases available in cohort 1, we used a predefined cutoff for signals of interest showing suggestive association of *P* < 10^–5^. After quality control filters were applied (see Methods), 502,663 SNPs from 48 cohort 1 hyponatremic cases on thiazides and 2,905 controls remained for association testing. The genomic inflation factor (λ = 1.007) and the resultant quantile-quantile (QQ) plot (Supplemental Figure 2) were not indicative of inflation of test statistics due to population substructure. In total, 17 SNPs within 14 regions were identified as showing suggestive association with TIH (*P* < 10^–5^) (Supplemental Figures 3 and 4 and Supplemental Table 6). Of these, we chose *SLCO2A1* for performing additional studies, given its potential role in altered prostaglandin transport and regulation of water reabsorption in the kidney.

In our GWAS data set, rs4854769, the intronic sentinel SNP within *SLCO2A1* showed association with TIH at *P* = 3.92 × 10^–6^ (odds ratio [OR] = 2.58). Targeted resequencing of *SLCO2A1* in cohort 1 TIH cases and cohort 1 controls confirmed the presence of the nonsynonymous variant encoding p.A396T (rs34550074) in complete linkage disequilibrium (*r^2^* = 1) with the sentinel GWAS SNP rs4854769. Association with rs34550074 between cohort 1 cases and the carefully phenotyped normonatremic cohort 1 controls on thiazide was observed at *P* = 0.0005 (OR = 3.3; Bonferroni’s corrected threshold with α 0.05 = 0.0017; Supplemental Table 7). The minor allele frequency (MAF) of rs34550074 in cohort 1 TIH cases was 0.35 (54% of TIH cases in cohort 1 carry at least 1 copy of the variant allele) compared with 0.14 in cohort 1 controls (25% carry at least 1 copy of the variant allele) and 0.18 in HAPMAP_CEU.

The total burden of rare protein-altering variants in other genes prioritized by the GWAS did not differ significantly between cohort 1 cases and controls after correcting for multiple testing (Supplemental Table 8). SLCO2A1 was nominally associated by the c-α test (*P* = 0.0019), driven by the association with p.A396T. Sanger sequencing also confirmed the presence of rs34550074 in cohort 1 hyponatremic TIH cases on thiazides (Supplemental Figure 5).

### Replication of association between SLCO2A1 p.A396T and TIH

The MAF for rs34550074 in cohort 2 TIH cases on thiazide was 0.26 (45% carried at least 1 copy of the variant allele) and 0.18 in normonatremic thiazide controls (test of association: *P* = 0.0304, OR = 1.70) (Supplemental Table 9). When data were combined across both cohorts, the pooled effect estimate for the association between rs34550074 and severe TIH was OR = 2.13 (*P* = 1.70 × 10^–4^, Supplemental Table 9).

### Prostaglandin transporter is expressed in the collecting duct of human cadaveric kidneys and colocalizes with AQP2

**Figure 3:**
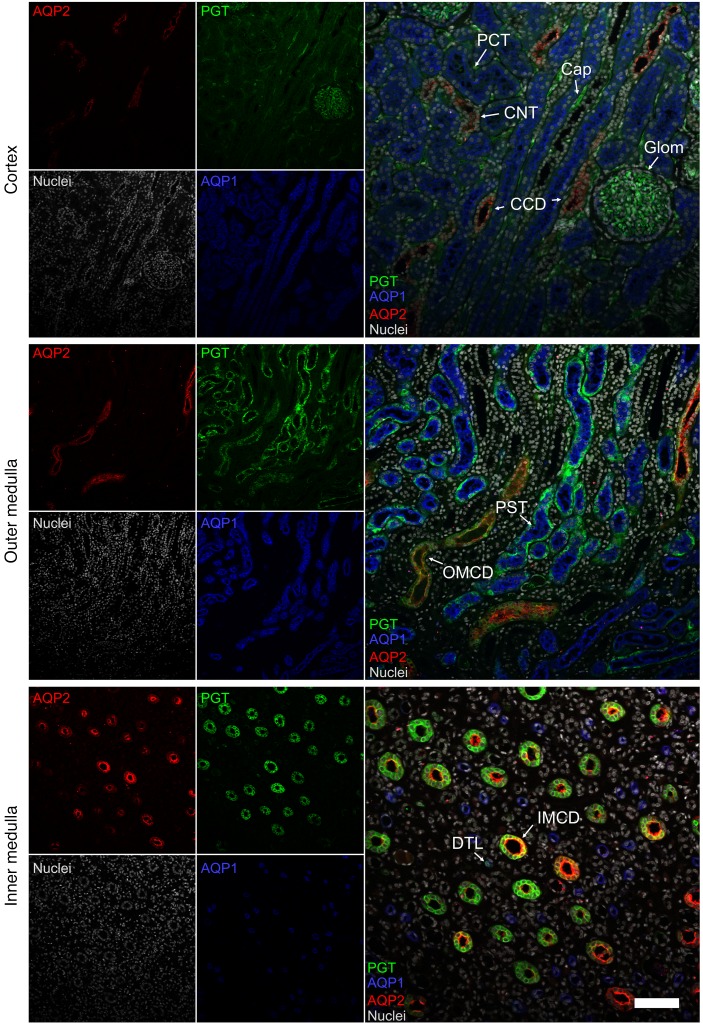
Renal expression of SLCO2A1 and colocalization with AQP1 and AQP2. Representative pseudocolored average intensity z projections of immunofluorescent-stained human cadaveric kidney sections showing the distribution of PGTs colocalized with aquaporin-1 (AQP1) and aquaporin-2 (AQP2). *n* = 8 biological replicates. Top panel: PGT is positive in glomeruli (Glom) and capillaries (Cap), but negative in AQP1-positive proximal convoluted tubules (PCT) and AQP2-positive connecting tubules (CNT) and cortical collecting ducts (CCD). Middle panel: PGT is found in the AQP1-positive proximal straight tubule (PST) and the AQP2-positive outer medullary collecting duct (OMCD). Bottom panel: AQP2-positive inner medullary collecting ducts (IMCD) stained strongly for PGT, with comparatively weak staining detectable in the AQP1-positive descending thin limb loop of Henle (DTL). Scale bar: 100 μm.

SLCO2A1 is principally expressed in the kidneys, adrenal glands, and lungs. Similarly to the renal cortex of the rat, human glomeruli and renal capillaries stained positive for prostaglandin transporter (PGT) (the protein product of *SLCO2A1*) (Figure 3 and Supplemental Figure 6, A and B) (20). Cortical tubules were primarily negative for PGT (Figure 3 and Supplemental Figure 6, C–E). PGT stained positive in the medulla, colocalizing with AQP1 and AQP2. Strong staining was detected in the proximal straight tubule, with comparatively weaker labeling of outer medullary collecting ducts and no PGT expression in the thick ascending limb loop of Henle (Figure 3 and Supplemental Figure 6, E–I). Expression of PGT increased as it transitioned into the inner medullary collecting ducts, with faint staining of the thin limb loop of Henle detectable only at higher laser/detector settings (Figure 3 and Supplemental Figure 6, J–M).

Species conservation of rs34550074 is shown in Supplemental Table 10. The tissue expression of genes near GWAS loci is shown in Supplemental Table 11.

### Urinary prostaglandin E_2_ and PGE_2_ metabolite concentrations are increased in TIH

Mean prostaglandin E_2_ (PGE_2_) and PGE_2_ metabolite (PGE_2_M) concentrations from 24-hour urine samples were significantly higher in cohort 2 hyponatremic TIH cases on thiazide compared with cohort 2 normonatremic thiazide controls (Supplemental Figure 7) and normalized after thiazide cessation. Analysis of urinary prostaglandin concentration by SLCO2A1 p.A396T status in cohort 2 demonstrated that TIH cases who carry at least 1 variant allele have significantly elevated urinary PGE_2_ and PGE_2_M concentrations relative to those homozygous for A396 (Figure 2, C and D). No such effect was observed in cohort 2 normonatremic thiazide controls.

### In vitro assessment of SLCO2A1 396 Ala/Thr/Glu variants

Figure 2E shows functional assays of PGE_2_ transport rate by 396Thr compared with that of 396Ala. The ratio of transport was not significantly different from 1. Because urinary PGE_2_ levels were higher in 396Thr compared with 396Ala subjects (Figure 2, C and D), we hypothesized that 396Thr may be subject to regulation by phosphorylation. Accordingly, we substituted Glu for Thr, since the charge and size will mimic phospho-Thr and recapitulate physiological effects of phosphorylation at a regulatory site. Figure 2E shows that the 396Glu moiety had a transport rate of 65% compared with 396Ala, suggesting that the 396Thr variant may result in reduced PGT function relative to 396Ala.

## Discussion

TIH remains a substantial clinical problem: population-based studies suggest that as many as 9% of subjects taking a thiazide may develop hyponatremia. Here, we report the largest and most detailed phenotypic description of TIH patients to date. In addition, we suggest a possible mechanism that may underlie TIH in some patients.

Systematic review of existing data suggests that TIH affects an older and predominantly female demographic with low serum osmolality, and limited spot urine testing suggests inappropriately concentrated urine and saliuresis (21). Our study supported this and also demonstrated a phenotype resembling SIADH, with low plasma osmolarity, inappropriately concentrated urine, more than minimal urinary sodium excretion, and normal thyroid function, but with low or normal ADH levels. Moreover, the phenotype of TIH also involved severe hypochloridemia, mild hyperglycemia, and intravascular volume expansion. Hypochloridemia and hypozincemia improved following thiazide cessation, but remained present 2 months after thiazide medication was stopped. Acutely hyponatremic TIH cases on thiazides demonstrated inappropriate net free water reabsorption and an exaggerated increase in free water reabsorption in response to thiazide exposure. This study therefore suggests there is much more to the phenotype of acute TIH than has been previously described. There are also phenotypic abnormalities that far outlast any kinetic or dynamic effect of thiazide medications, raising the possibility that such features might be present at baseline before thiazide commencement.

Having defined the phenotype of TIH, we set out to identify possible genetic predisposition to this condition. Given the small number of cases available in our initial cohort, we chose to prioritize GWAS associations with suggestive evidence for follow-up using a less conservative *P* value cut off (*P* < 10^–5^). We focused on *SLCO2A1* (encoding PGT) because augmented renal water reabsorption seemed most relevant to the principal abnormality of hyponatremia from our phenotype studies. PGT is known to regulate this process, and there was direct association between a protein-altering variant in SLCO2A1 (p.A396T) and TIH. This association was replicated in a second independent cohort. SLCO2A1 p.A396T was twice as frequent in TIH cases compared with control and general populations, with approximately half of all TIH patients carrying this variant. In vitro data suggest that this variant may reduce PGT activity and lead to the higher urinary PGE_2_ concentrations observed in TIH patients who carry the variant allele. However, since half of TIH patients do not carry the *SLCO2A1* variant and at least a quarter of unaffected patients do, other genetic or environmental determinants must also contribute to the development of TIH.

One potential criticism of this study is the limited sample size in the discovery cohort. This was inevitable, since we were careful to include only those with severe thiazide-induced hyponatremia and excluded those who lacked mental capacity or who had relevant comorbidities.

**Figure 4:**
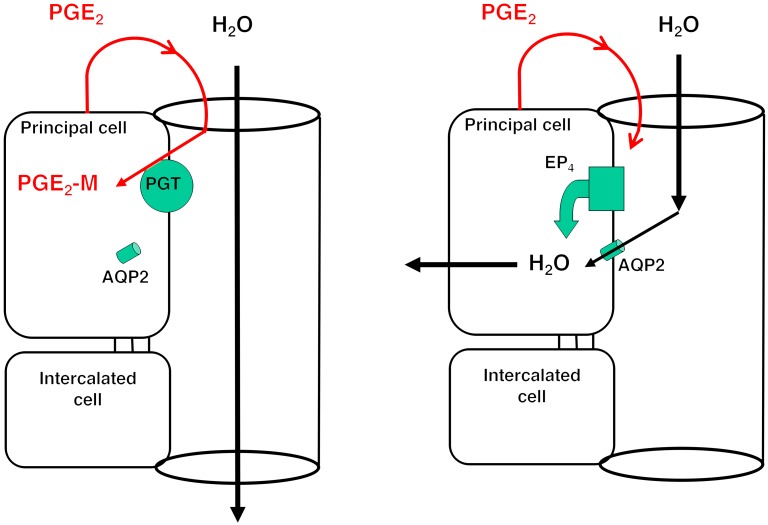
Hypothesis for the role of SLCO2A1 in contributing to TIH in individuals carrying the SLCO2A1 A396T variant. (**A**) Under low ADH conditions, apical PGT in the renal collecting duct scavenges PGE_2_ from the lumen, resulting in AQP2 internalization and minimal osmotic water reabsorption. (**B**) With reduced or absent apical PGT, PGE_2_ reaching the lumen is able to stimulate apical EP4 receptors, resulting in insertion of AQP2 and osmotic water reabsorption.

So how does one reconcile suppressed ADH with elevated PGE_2_ and intravascular volume expansion? And how might a reduction in SLCO2A1 activity cause TIH? SLCO2A1 is expressed at the apical membrane of the renal collecting duct, where it scavenges newly synthesized PGE_2_ away from luminal EP_4_ receptors (22, 23). Activation of these apical EP4 receptors increases the water permeability of this nephron segment 10- to 15-fold, even in the absence of ADH (24, 25). Thus, reduced activity of collecting duct apical SLCO2A1 would be predicted to increase hydraulic conductivity of the collecting duct, even in the absence of ADH (Figure 4). Although urinary osmolarity in the TIH subjects was 15% higher than in control subjects (Supplemental Table 3) (probably as a result of increased renal medullary PGE_2_; ref. 26), which would mitigate water reabsorption, the proportionally larger increase in collecting duct water permeability observed would more than offset the slight reduction in osmotic driving force.

Although the putative effects of SLCO2A1 inactivation appear to be compensated under normal conditions, they are made manifest when the patients are given a thiazide diuretic. Thiazides reduce the ability of the late diluting segment (DCT) to generate solute-free water directly and also act by reducing effective vascular volume and thus solute delivery from the end-proximal tubule (27). We suggest therefore that, in individuals carrying the SLCO2A1 A396T variant, the combination of thiazide-specific effects on free water generation and the increase in collecting duct water permeability from reduced SLCO2A1 activity combine to produce TIH (Figure 4).

## Methods

### Clinical recruitment.

Two cohorts of patients of mixed European descent hospitalized with symptomatic TIH were recruited (Figure 1 and Supplemental Methods). Cohort 1 comprised 48 hyponatremic TIH cases on thiazides (serum sodium < 130 mM) recruited during their acute admission to Nottingham University Hospitals NHS Trust and matched healthy normonatremic thiazide controls from primary care. Cohort 2 comprised a further 109 hyponatremic TIH cases on thiazides recruited during their acute admission to the same hospital together with 2 matched control groups from primary care (cohort 2 normonatremic thiazide controls and cohort 2 normonatremic nonthiazide controls). Cohort 2 TIH cases were also assessed 2 months after thiazide cessation (termed cohort 2 normonatremic TIH cases off thiazides).

### GWAS.

A GWAS was performed using 48 cohort 1 TIH cases genotyped using the Illumina Omni1quad array and 2,922 general population controls from the British 1958 birth cohort (28), genotyped using the Illumina 1.2M chip. Controls were all aged 44 to 45 years at the time of DNA collection (2002 to 2004) and 48% were female. Following quality control of the genotype data (Supplemental Methods), 502,663 SNPs that were genotyped in both cases and controls remained for association testing. A case-control association analysis was undertaken using a logistic regression model, with adjustment for 10 principal components and assuming an additive genetic model, using Plink v 1.07.

### Resequencing and replication studies.

101 samples (48 cohort 1 hyponatremic TIH cases on thiazides and 53 cohort 1 normonatremic thiazide controls) underwent resequencing of the genes nearest to SNPs that were associated with TIH at *P* < 10^–5^, followed by association testing (Supplemental Table 7 and Supplemental Methods). Next Generation sequencing data were deposited at the European Nucleotide Archive (ENA accession number PRJEB21924; http://www.ebi.ac.uk/ena). Replication for one SNP of interest identified in the sequencing analysis of cohort 1 (rs34550074, p.A396T) was undertaken using Sanger sequencing in the second cohort of TIH cases and controls (Supplemental Methods). Rare variant association was carried out using Plink/Seq (29). Rare variants were assessed using the burden test, which is a collapsing allelic sum test using adaptive permutation to derive an empirical *P* value. Combined rare and common variants were also assessed using the c-α test (30), also implemented in Plink/Seq.

### Kidney immunofluorescence.

Formalin-fixed paraffin-embedded human tissue sections were obtained from the Cambridge Human Research Tissue Bank. After antibody labeling, confocal imaging was performed (Supplemental Methods).

### In vitro functional studies of SLCO2A1 p.A396T.

Human SLCO2A1 cDNA (encoding PGT) was modified by site-directed mutagenesis using standard methods so as to generate cDNAs encoding amino acids Ala, Thr, or Glu at position 396. DNA sequences were confirmed by direct sequencing. Human embryonic kidney (HEK) cells were transiently transfected with one of the 3 cDNAs, and the timed uptake of ^3^H-PGE_2_ was assayed as described previously (26).

### Statistics.

Significance between groups was assessed using a 2-tailed Student’s *t* test. Where comparisons between more than 2 groups were undertaken, 1-way ANOVA and Bonferroni’s post hoc test were used. The data were analyzed using Graph Pad Prism V6.05. *P* < 0.05 was considered significant. Statistical methods used for GWAS and resequencing experiments are contained within the relevant Methods and Supplemental Methods sections.

### Study approval.

This study was conducted in line with the standards of ICH/Good Clinical Practise sections 8.2.8 and was given approval by the Queen’s Medical Centre Ethics Committee (cohort 1; reference GM030208) and the UK National Research Ethics Committee (cohort 2; reference 11/EM/0233). Written informed consent was received from participants prior to inclusion in the study.

## Author contributions

JSW, SC, LVW, SKC, VEJ, EE, RL, KS, WJ, NS, APH, and MG performed experiments and analyzed data. SK, MJ, JC, and MG recruited patients. KMO, VLS, MDT, IPH, and MG designed the study. All authors contributed to the drafting and revision of the manuscript, which was led by MG and IPH.

## Supplementary Material

Supplemental data

## Figures and Tables

**Table 1:**
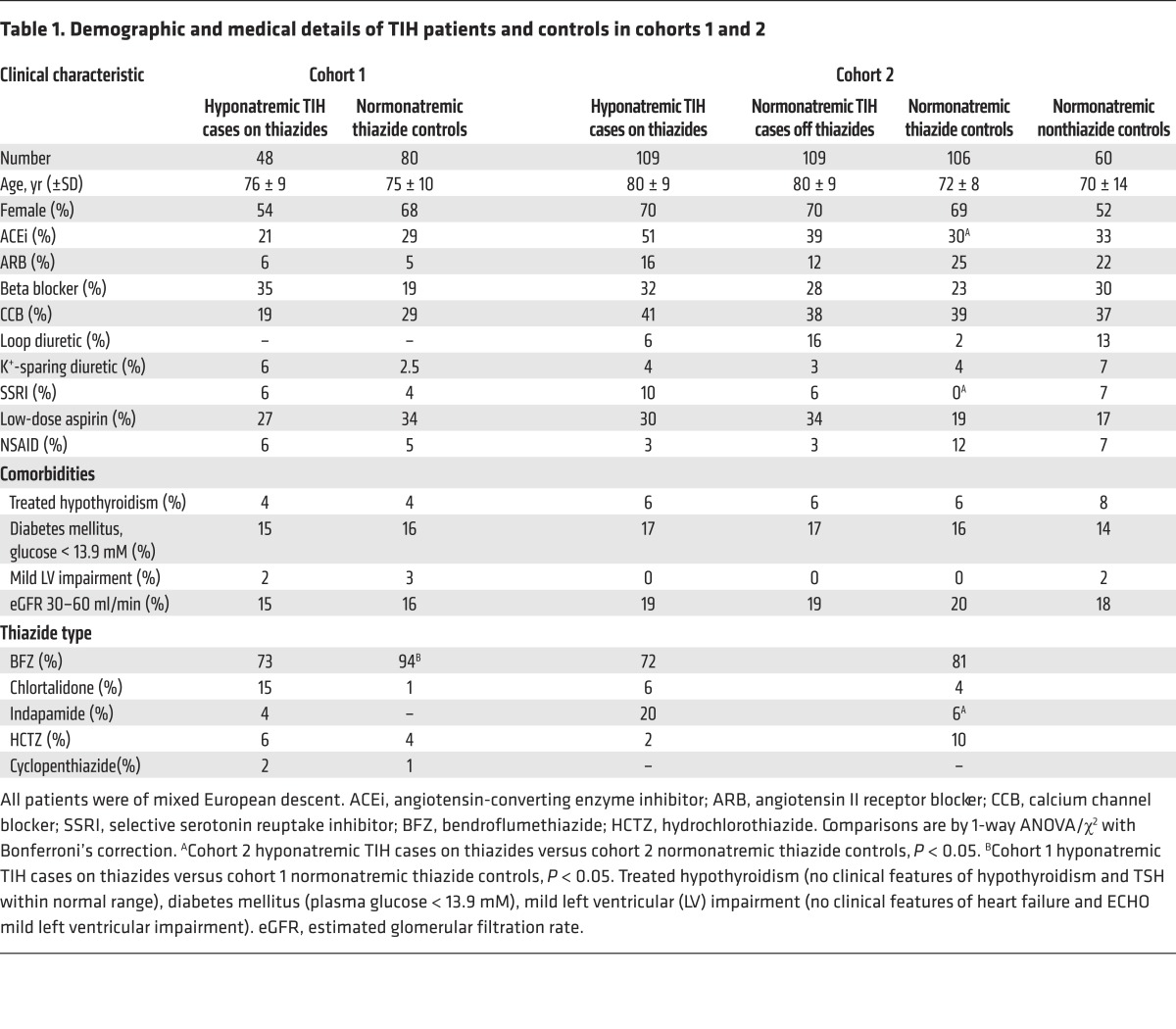
Demographic and medical details of TIH patients and controls in cohorts 1 and 2

## Version 1 08/07/2017

Electronic publication

## Version 2 09/01/2017

Print issue publication
