# Supplementary material for: Phenotypic and pharmacogenetic evaluation of patients with thiazide-induced hyponatremia
Source: J Clin Invest. 2017 Aug 7;127(9):3367–74. doi: 10.1172/JCI89812 (PMC5669583; doi:10.1172/JCI89812)
Supplement: Supplemental data [file jci-127-89812-s001.pdf]

Supplementary Materials

Index **Page**

Supplementary Methods

Clinical recruitment 3

Phenotypic studies of blood and urine samples 4

Measurement of fractional uric acid clearance 6

Plasma ADH measurement 6

Measurement of urinary PGE_2 and PGE_2 Metabolite (PGE_2M) concentration 6

Genetic studies 7

Genome Wide Association study 7

Resequencing of GWAS candidate regions 7

Kidney immunofluorescence 9

Supplementary results

Phenotype of TIH cases and controls 10

Results of genetic studies 14

Genome Wide Association Study of cohort 1 TIH cases and

Controls from the 1958 birth cohort 14

Interrogation of GWAS candidate regions 21

Human kidney immunofluorescence- PGT kidney expression 26-31

Urinary PGE_2 and PGE_2 Metabolite (PGE_2M) concentration 30-31

Supplementary Tables

Supplementary Table S1 5

Supplementary Table S2 11

Supplementary Table S3 12

Supplementary Table S4 13

Supplementary Table S5 15

Supplementary Table S6 17

Supplementary Table S7 21

Supplementary Table S8 22

Supplementary Table S9 24

Supplementary table 10 25

Supplementary table 11 26

Supplementary Figures

Supplementary Figure S1 14

Supplementary Figure S2 16

Supplementary Figure S3 18

Supplementary Figure S4 18-20

Supplementary Figure S5 24

Supplementary Figure S6 27-31

Supplementary Figure S7 32

Supplementary methods

Clinical recruitment

Cohort 1; This study was conducted in line with the standards of ICH/Good Clinical Practise sections 8.2.8 and was given approval by the Queen’s Medical Centre Ethics committee (study approval reference GM030208). Between July 2002 and November 2003 a daily search of the biochemistry data base of all patients admitted to the department of internal medicine at Nottingham University Hospitals NHS Trust, UK was undertaken to identify those patients with serum sodium <130mM (cases) and those with serum sodium concentration 135-145mM (controls). Inclusion criteria were to be a hospital inpatient aged sixteen to ninety years taking any thiazide or thiazide-like diuretic. For hyponatremic TIH cases on thiazides, the clinical diagnosis of thiazide-induced hyponatremia without any other cause of hyponatremia was necessary. Specifically those with cirrhosis, end stage heart failure, renal impairment (eGFR<30ml/min), nephrotic syndrome, pregnancy, syndrome of inappropriate anti-diuretic hormone secretion, untreated endocrine disease (hypothyroidism, hypopituitarism, Addison’s disease), diarrhoea, vomiting, clinical dehydration, hyperlipidemia, hyperproteinemia, blood glucose>13.9mM, clofibrate, carbamazepine, chlorpropramide, anti-neoplastic drugs or any other cause of hyponatremia apart from a thiazide or thiazide-like diuretic evident to the treating clinician or research team were excluded. Patients read a study information sheet and informed consent was taken. Clinical biochemistry results were recorded from the hospital computer system and a single 8ml EDTA blood sample taken for DNA extraction.

Cohort 2; hypoantremic TIH cases on thiazides were identified in the same way as cohort 1 between April 2012 and August 2015 (UK national research ethics committee reference 11/EM/0233). Inclusion criteria were age 18-100 years with capacity to give informed consent, taking a thiazide or thiazide-related diuretic (except control group 2), blood sodium concentration of less than 130 mM for cases or sodium between 135 mM and 145 mM for

controls. Exclusion criteria for cases and controls were patients who lack capacity to consent, those who have an alternative medical cause (other than the thiazide) for their hyponatremia including but not limited to: cirrhosis or other liver failure, severe heart failure, severe renal failure (eGFR < 30 ml/ minute) or nephrotic syndrome, pregnancy, untreated endocrine diseases including but not limited to hypothyroidism, hypoadrenalisism and hypopituitarism, Syndrome of Inappropriate Antidiuretic secretion (SIADH), hyperglycemia (glucose greater than 13.9 mM). After informed consent was obtained an 8ml EDTA blood sample taken for DNA, and an additional 11ml serum and 8ml plasma was also collected for phenotypic studies together with commencement of plain 24 hour urine collection and a single 20ml spot urine sample with protease inhibitor (cOmplete, Mini, EDTA-free®- Roche) so long as intravenous fluids had not been administered by the regular care team. Hyponatremic TIH cases were then assessed in the hypertension outpatient clinic after two months without thiazide and repeat blood and urine samples taken. Normonatremic thiazide and non-thiazide controls (serum sodium 135-145 mM) were identified by primary care surgeries in Nottinghamshire and matched as closely as possible to the cases for age, sex, comorbidities and polypharmacy.

Phenotypic studies of blood and urine samples

In cohort 2 serum, plasma and 24h urine were analysed for a range of physiological parameters by the biochemistry department at Nottingham University Hospitals NHS Trust using the same methods as for routine clinical care (Supplementary Table 1).

Biochemistry parameter	Method	Machine
Serum:		
**Sodium**	Indirect ion selective electrode	Beckman AU clinical chemistry analyser
**Potassium**	Indirect ion selective electrode	Beckman AU clinical chemistry analyser
**Urea**	GLDH kinetic UV test method	Beckman AU clinical chemistry analyser
**Creatinine**	IDMS-traceable kinetic colour test (Jaffe) method	Beckman AU clinical chemistry analyser
**Osmolarity**	Freezing point depression	3300 Micro-Osmometer
**TSH**	Sandwich immunoassay	Siemens Advia Centaur
**Glucose**	Hexokinase UV method	Beckman AU clinical chemistry analyser
**Chloride**	Indirect ion selective electrode	Beckman AU clinical chemistry analyser
**corrected Calcium**	Arsenazo III Photometric colorimetric test method	Beckman AU clinical chemistry analyser
**Phosphate**	Photometric UV test method	Beckman AU clinical chemistry analyser
**Magnesium**	Colorimetric test method	Beckman AU clinical chemistry analyser
**Bicarbonate**	Colorimetric enzymatic assay	Beckman AU clinical chemistry analyser
**Zinc**	ICP-MS	Agilent 7700x ICP-MS
**Vitamin D**	Immunoassay	Siemens Advia Centaur
Plasma:		
**Renin**	Chemiluminescent immunoassay	DiaSorin analyser
**Aldosterone**	Chemiluminescent immunoassay	DiaSorin analyser
**PTH**	Immunoassay	Siemens Advia Centaur
24h urine:		
**Sodium**	Indirect ion selective electrode	Beckman AU clinical chemistry analyser
**Potassium**	Indirect ion selective electrode	Beckman AU clinical chemistry analyser
**Urea**	GLDH kinetic UV test method	Beckman AU clinical chemistry analyser
**Creatinine**	IDMS-traceable kinetic colour test (Jaffe) method	Beckman AU clinical chemistry analyser
**Chloride**	Indirect ion selective electrode	Beckman AU clinical chemistry analyser
**Calcium**	Arsenazo III Photometric colorimetric test method	Beckman AU clinical chemistry analyser
**Phosphate**	Photometric UV test method	Beckman AU clinical chemistry analyser
**Magnesium**	Colorimetric test method	Beckman AU clinical chemistry analyser

Supplementary Table S1. Parameters and methods of blood and urine analysis of TIH and control patients by Nottingham University Hospitals NHS Trust biochemistry department.

Measurement of fractional uric acid clearance

Serum and 24h urine samples were analysed for uric acid using the uric acid colorimetric kit (Bio Vision, USA). In brief, serum and urine samples were diluted in uric acid assay buffer. Reaction mix was added to samples and standards. Absorbance (OD) was measured using FlexStation® 3 Multi-Mode Microplate Reader (Molecular Devices Corporation, U.S.A.) at 570nm. Several dilutions were tested to ensure readings are within the standards range and the assay was carried in duplicates at room temperature. Creatinine clearance (Ccl) was calculated from the formula Ccl = Uv x Ucr/Scr, expressed in ml/ min (where Uv is urine volume/24hrs, Ucr is urinary creatinine, and Scr is serum creatinine). Uric acid clearance (UAcl) was calculated from the formula UAcl = Uv x Uua/Sua, expressed in ml/min (where Uua is urinary uric acid and Sua is serum uric acid concentration). Fractional uric acid clearance (FUAcl) was calculated as FUAcl = UAcl/Ccl x 100, and expressed as a percentage.

Plasma ADH measurement

Plasma samples were analysed for ADH using the Arg8-vasopressin ELISA kit (Abcam, UK). In brief, plasma samples were diluted in assay buffer and assay was carried in duplicates at room temperature. Several dilutions were tested to ensure readings are within the standards range. Absorbance (OD) was measured using FlexStation® 3 Multi-Mode Microplate Reader (Molecular Devices Corporation, U.S.A.) at 405nm. Samples within 20-80% percentage bound were considered for further analysis, taking in to account appropriate dilution factors.

Measurement of urinary PGE2 and PGE2 Metabolite (PGE2M) concentration

Urinary PGE2 and PGE2M levels were measured at room temperature by a commercialized enzyme linked immunosorbent assay (Prostaglandin E2 and Prostaglandin E Metabolite EIA

kits, Cayman Chemical, UK). For PGE2 analysis, Standard (10 ng/ml) was used to produce a dilution series (2500 pg/ml set as highest standard). For PGE2M analysis, Standard (1000 pg/ml) was used to produce a dilution series (50 pg/ml set as highest standard). The microplate was prepared with calibrators, standards and sample according to manufacturer's instructions. The optical density of each well was determined within 10 minutes, using a FlexStation® 3 Multi-Mode Microplate Reader (Molecular Devices Corporation, U.S.A.) at 420 nm. Several dilutions were tested to ensure readings are within the standards range and each sample was carried out in duplicate. Samples within 20-80% percentage bound were considered for further analysis and data was normalized using urinary creatinine and 24h urine volume.

<u>Genetic studies</u>

Genome Wide Association Study

Quality control of the genotype data was carried out separately in the case and control datasets. Individuals with >5% missing genotype data were excluded. SNPs were excluded if they had more than 5% data missing, had a minor allele frequency (MAF) less than 1%, or if they significantly deviated from Hardy Weinberg Equilibrium (HWE) at the P=0.001 level. A/T and C/G SNPs were additionally excluded due to strand assignment issues, along with SNPs which were identified as having differential missingness in cases and controls (number of missing genotypes significantly differed in cases and controls at the P=0.01 level). Association testing was carried out using Plink v 1.07, using a logistic regression model, with adjustment for 10 principal components and assuming an additive genetic model. Post-association testing, cluster plots were generated for all SNPs found to be significant at the $P<1.0x10^{-5}$ level, and checked for incorrect genotype calling.

Resequencing of GWAS candidate regions

Tiled PCR amplicons were prepared to cover all exons and splice junctions from genomic DNA using a microfluidic PCR system (Fluidigm Access Array) followed by sequencing on the Illumina HiSeq. Reads were aligned using bwa (v0.7.5a-r416), and GATK (v2.8-1) was

used for base recalibration, local realignment, and multi-sample variant calling using Unified Genotyper, according to best practice guidelines. GATK variant filter parameters used for sequencing were: (a) for SOLiD data: SNPs:DP < 4, GQ < 5, FS > 60.0, MQ < 40.0, MQRankSum < -12.5, QD < 2.0, ReadPosRankSum < -8.0 Indels: DP<4, GQ<5, QD < 2.0, ReadPosRankSum < -20.0, InbreedingCoeff < -0.8, FS>200.0. (b) for Illumina data: SNPs: DP<4, GQ<5, QD < 3.0, MQ < 30.0 Indel: DP<4, GQ<5, QD < 3.0. Variants were annotated using SnpEff (v3.3; using ensembl canonical transcripts vGRCh37.73), and SnpSift (v3.3h; using dbSNPv138 and dbNSFPv2.0). All samples passed quality control metrics and were included in analyses. Variants with missing genotypes in more than 5 samples, or deviating from HWE in controls were removed.

Association tests were performed using PLINK/SEQ (v0.08-x86_64). To identify coding variation underlying the suggestive GWAS associations, for each candidate gene, the coding SNP with the strongest association was identified. Fisher's exact test was used to perform allelic single variant tests on protein-altering variants with a combined allele count of at least 7 across the population (an allele count powered to give a nominal p-value < 0.005 if all variant alleles were restricted to cases). Gene-wise burdens of protein-altering variation were also assessed both by collapsing rare variants (MAF < 0.01 and then to MAF < 0.001), and by applying c-alpha to all variants.

Replication of rs34550074 (p.A396T) in cohort 2 TIH cases and controls

94 TIH cases and 106 normonatremic thiazide controls underwent Sanger sequencing at rs34550074 (p.A396T). A case-control association analysis was undertaken using a Fisher's Exact Test (allelic model), The results for both the Cohort 1 and Cohort 2 TIH cases normonatremic thiazide controls were combined using a Cochran-Mantel-Haenszel meta-analysis to give an overall estimated effect.

Reads were aligned and variants identified and annotated using the pipeline described above for GWAS loci resequencing, but with specific parameters appropriate for the target enrichment and sequencing platform. (GATK variant filters - SNPs: DP < 4, GQ < 5, FS > 60.0, MQ < 40.0, MQRankSum < -12.5, QD < 2.0, ReadPosRankSum < -8.0 Indels: DP<4, GQ<5, QD < 2.0, ReadPosRankSum < -20.0, InbreedingCoeff < -0.8, FS>200.0). Variants with missing genotypes in more than 5 samples or that deviated from HWE in controls were removed as before. Three samples with poor sequencing coverage were excluded from analyses.

Fisher's exact test was used to perform additive single variant tests on protein-altering variants with a combined allele count of at least 8 across the population (an allele count powered to give a nominal p-value < 0.005 if variant alleles restricted to cases). Gene-wise burdens of protein-altering variation were assessed both by collapsing rare variants (MAF < 0.01 or 0.001), and by applying c-alpha to all variants.

Kidney Immunofluorescence

Formalin-fixed paraffin embedded human tissue sections were obtained from the Cambridge Human Research Tissue Bank. 5 µm sections were deparaffinised in Histoclear (National Diagnostics) and rehydrated in graded methanol steps. An antigen retrieval step was performed with R-Universal buffer in the 2100 antigen retriever for a single heat-pressure cycle (Aptum Biologics). Sections were permeabilized with 0.05% v/v Triton-X100-PBS for 20 mins and blocked for 1 h at 37 °C with 2% v/v donkey serum in 0.05% v/v Triton-X100-PBS. Primary antibodies were incubated overnight for 16 h at 4 °C at the following concentrations diluted in 1% v/v donkey serum in 0.05% v/v Triton-X100-PBS: 2 µg/mL rabbit anti-PGT (11860; Cayman Chemical), sheep anti-NCC (S965B, MRC-PPU Reagents), sheep anti-NKCC2 antibody (S838B, MRC-PPU Reagents); 1:100 mouse anti-AQP1 (ab9566; Abcam); 1:2000 goat anti-AQP2 (sc-9882; Santa Cruz Biotechnology). Slides were then washed for 20 mins in 0.05% v/v Triton-X100-PBS and incubated in secondary antibody

for 1 h at 37 °C. Pre-absorbed donkey IgG conjugated Alexa Fluor 488, 555, 633 and 647 secondary antibodies (Life Technologies/Abcam) were used at 1:200 diluted in 1% v/v donkey serum in 0.05% v/v Triton-X100-PBS for immunofluorescent labelling. Slides were washed as above, followed by a 5 mins wash in distilled water before counterstaining of nuclei with 1:1000 Sytox Blue (S34857, Life Technologies) for 30 mins at room temperature and again washed for 5 mins in distilled water. Finally slides were mounted using Prolong gold antifade (P36930, Life Technologies) and shielded from light until imaging.

Immunofluorescent images were acquired on the Leica TCS SP2 laser-scanning confocal with 458nm, 488 nm, 543 nm, 633 nm laser lines mounted on an upright Leica DM RXA fluorescent microscope using either a HC PL FLUOTAR 10X/0.3NA or HC PL FLUOTAR 20X/0.5NA dry objective. Acquisition parameters: 8-bit, 1024x1024 pixels, between 1 – 3X digital zoom, 400 Hz scan speed, 4-line Kalman filtering, sequential (by line) channel imaging, 10 slice z-stack of 5µm. In FIJI image analysis software (fiji.sc/Fiji), fluorescent z-stacks underwent background subtraction (1000 pixel radius rolling ball, no smoothing) and average intensity z-projection. Image brightness and contrast were uniformly adjusted for each individual image by linear histogram stretching to enhance visibility.

Supplementary Results

Phenotype of TIH cases and controls

	Cohort 1		Cohort 2			
	Hyponatremic TIH cases on thiazide n=48	Normonatremic thiazide controls n=80	Hyponatremic TIH cases on thiazides n=109	Normonatremic TIH cases off thiazides n=109	Normonatremic thiazide controls n=106	Normonatremic non-thiazide controls n=60
S.Sodium mM	118 +/-0.9	139 +/-0.3****	122+/-0.6	137+/-0.4††††	139 +/-0.2¶¶¶¶	139+/-0.3
S.Potassium mM	3.7 +/-0.09	4.0 +/-0.06 **	3.7 +/-0.06	4.4+/-0.05††††	3.9+/-0.03	4.1+/-0.07§
S. Urea mM	6.0 +/-0.4	7.7 +/-0.3	7.7 +/-0.6	6.3+/-0.3	6.5+/-0.2	7.6+/-1.5
S. Creatinine µM	79 +/-5	94 +/-4*	80 +/-3	74+/-2	82+/-2	80+/-3
S.Osmolarity mosmol/Kg	248 +/-2	289+/-1****	255 +/-4	288+/-1††††	295+/-1¶¶¶¶	293+/-1
eGFR ml/min			75+/-2	73+/-2	68+/-1	73+/-2
TSH mU/L			2.1+/-0.3	2.7+/-0.3	2.5+/-0.2	2.1+/-0.2
Glucose mM	6.8+/-0.3	6.6+/-0.2	7.6+/-0.4	5.9+/-0.2††	6.1+/-0.2¶¶	6.5+/-0.4
S. Chloride mM			89+/-0.9	99+/-0.4††††	100+/-0.3¶¶¶¶	102+/-0.5§§
S. corrected Calcium mM			2.34+/-0.02	2.39+/-0.01	2.41+/-0.01¶¶	2.31+/-0.02§§
S. Phosphate			1.02 +/-0.03	1.17+/-0.02††††	1.08+/-0.02	1.06+/-0.04§§
S. Magnesium mM			0.75+/-0.02	0.81+/-0.01††	0.80+/-0.01¶	0.84+/-0.01
S. Bicarbonate mM			26.4+/-0.6	28.0+/-0.4	26.8+/-0.3	26.7+/-0.5
S. Zinc µM			9.4+/-0.3	10.6+/-0.2	12.6+/-0.3¶¶¶¶	12.3+/-0.2§§
P. Renin mU/L			133+/-34	69+/-20	86+/-14	57+/-18
P. Renin mU/L excluding those taking a β blocker			148+/-43	83+/-25	92+/-17	74+/-26
P. Aldosterone pM			328+/-45	255+/-20	346+/-21	293+/-29
S. Vitamin D nM			34+/-3	38+/-2	45+/-2*	33+/-4
P. PTH ng/L			49+/-4	64+/-4	54+/-2	72+/-6
Systolic BP mmHg			139 +/-2.7	157+/-2.7††††	152+/-1.9¶¶¶	147 +/- 3.5
Diastolic BP mmHg			72 +/-1.1	80 +/-1.3††††	82+/-1.1¶¶¶¶	82+/-1.8
BMI (Kg m^{-2})			25.7+/-0.6	25.4+/-0.5	24.1+/-0.5	27.2+/-0.6
Weight (Kg)			66.3+/-1.7	65.5+/-2.1	81.1+/-1.7¶¶¶¶	79.0+/-2.4§§§

Supplementary table S2 – Phenotype of TIH cases and controls in cohorts 1 and 2. 'S.' (Serum), 'P.' (Plasma), estimated Glomerular Filtration Rate (eGFR), Thyroid Stimulating Hormone (TSH), ParaThyroid Hormone (PTH), Body Mass Index (BMI). Comparisons are by 1 way ANOVA with Bonferroni correction. * cohort 1 hyponatremic TIH cases on thiazides vs cohort 1 normonatremic thiazide controls: *$p<0.05$, ** $p < 0.005$ ***$p<10^{-4}$, ****$p<10^{-5}$. † cohort 2 hyponatremic TIH cases on thiazides vs cohort 2 normonatremic TIH cases off thiazides: † $p<0.05$, †† $p < 0.005$ $^{\dagger\dagger\dagger}p<10^{-4}$, $^{\dagger\dagger\dagger\dagger}p<10^{-5}$.¶ cohort 2 hyponatremic TIH cases on thiazides vs cohort 2 normonatremic thiazide controls: ¶ $p<0.05$, ¶¶ $p < 0.005$ $^{\P\P\P}p<10^{-4}$, $^{\P\P\P\P}p<10^{-5}$.§ cohort 2 normonatremic non-thiazide controls vs cohort 2 normonatremic TIH cases off thiazides: § $p<0.05$, §§ $p < 0.005$ $^{\S\S\S}p<10^{-4}$, $^{\S\S\S\S}p<10^{-5}$.

Biochemistry Characteristic	Cohort 1		Cohort 2 cases		Cohort 2 controls	
	Hyponatremic TIH cases on thiazide n=48	Normonatremic thiazide controls n=80	Hyponatremic TIH cases on thiazides n=109	Normonatremic TIH cases off thiazides n=109	Normonatremic thiazide controls n=106	Normonatremic non-thiazide controls n=60
Spot U.Osmolarity mosmol/Kg	465	-	366+/-	-		
Spot U. Sodium mM	23	-	31+/-	-		
Spot U. Potassium mM	-	-	39 +/-	-		
24h volume ml			1305+/-167	1571+/-72	1671 +/-42[¶]	1700+/-72
24h urine osmolarity mosmol/Kg			352+/-23	351+/-14	438 +/-15[¶]	415+/-22
24h urine creatinine mM			5+/-0.6	5+/-0.2	7+/-0.4	6+/-0.4
24h urine creatinine mmoles			5+/-0.5	6+/-0.4	10+/-0.4[¶¶¶¶]	10+/-0.4[§§§§]
24h urine sodium mM			39 +/-4	55+/-3[†]	67 +/-3[¶¶¶¶]	59+/-3
24h urine sodium mmoles			47+/-6	80+/-5[††]	109 +/-5[¶¶¶¶]	98+/-7
24h urine sodium/creatinine			10 +/-1	13+/-1[†]	11+/-0	10+/-1[§]
24h urine potassium mM			29+/-3	34+/-1	47+/-1[¶¶¶¶]	44+/-2[§§]
24h urine potassium mmoles			30+/-3	50+/-2[††††]	74+/-2[¶¶¶¶]	72+/-4[§§§§]
24h urine potassium/creatinine			6+/-1	8+/-0[††]	8+/-0[¶¶]	8+/-0
24h urine urea mM			165+/-13	147+/-8	200+/-7	200+/-12[§§]
24h urine urea mmoles			170+/-15	206+/-10	319+/-10[¶¶¶¶]	309+/-15[§§§§]
24h urine urea/creatinine			34+/-2	34+/-1	32+/-1	29+/-2
24h urine chloride mM			40+/4	59+/-3[††]	71+/-3[¶¶¶¶]	65+/-4
24h urine chloride mmoles			46+/-6	88+/-6[†††]	114+/-5[¶¶¶¶]	107+/-7
24h urine chloride/creatinine			10+/-1	14+/-1[††]	12+/-0	11+/-1[§]
24h urine calcium mM			1.74+/-0.21	1.83+/-0.15	1.89+/-0.13	2.19+/-0.24
24h urine calcium mmoles			2.03+/-0.32	2.56+/-0.22	3.01+/-0.20	3.68+/-0.45[§]
24h urine calcium/creatinine			0.38+/-0.53	0.42+/-0.03	0.31+/-0.02	0.37+/-0.05
24h urine magnesium mM			1.24+/-0.16	1.68+/-0.11	2.25+/-0.10[¶¶¶¶]	2.22+/-0.17[§]
24h urine magnesium mmoles			1.46+/-0.24	2.39+/-0.15[†]	3.60+/-0.15[¶¶¶¶]	3.58+/-0.24[§§§]
24h urine magnesium/creatinine			0.32+/-0.04	0.39+/-0.02	0.38+/-0.01	0.38+/-0.02
24h urine phosphate mM			8.0+/-1.1	10.5+/-0.6	14.1+/-0.5[¶¶¶¶]	14.4+/-0.8[§§]
24h urine phosphate mmoles			8.4+/-1.2	15.3+/-0.9[†††]	22.8+/-0.8 [¶¶¶¶]	23.1+/-1.1[§§§§]
24h urine phosphate/creatinine			1.50+/-0.16	2.39+/-0.08[††††]	2.36+/-0.08[¶¶¶¶]	2.45+/-0.08
24h urine zinc µM			8.1+/-1.1	3.7+/-0.3[††††]	5.2+/-0.4[¶¶]	4.8+/-0.7
24h urine zinc µmoles			8.2+/-1.1	5.6+/-0.5	8.3+/-0.6	7.6+/-0.9
24h urine zinc/creatinine			1.52+/-0.16	1.01+/-0.15	0.87+/-0.09	0.75+/-0.06
Free water reabsorption ml/min			0.34+/-0.01	0.23+/-0.01	0.51+/-0.05	0.47+/-0.02[§§]

Supplementary table S3. Description of urinary phenotype of TIH cases and controls in cohorts 1 and 2. 1 way ANOVA with Bonferroni correction. * cohort 1 hyponatremic TIH cases on thiazides vs cohort 1 normonatremic thiazide controls: *p<0.05, ** p < 0.005 ***$p<10^{-4}$, ****$p<10^{-5}$. † cohort 2 hyponatremic TIH cases on thiazides vs cohort 2 normonatremic TIH cases off thiazides: † p<0.05, †† p < 0.005 †††$p<10^{-4}$, ††††$p<10^{-5}$.¶ cohort 2 hyponatremic TIH cases on thiazides vs cohort 2 normonatremic thiazide controls: ¶ p<0.05, ¶¶ p < 0.005 ¶¶¶$p<10^{-4}$, ¶¶¶¶$p<10^{-5}$.§ cohort 2 normonatremic non-thiazide controls vs cohort 2 normonatremic TIH cases off thiazides: § p<0.05, §§ p < 0.005 §§§$p<10^{-4}$, §§§§$p<10^{-5}$. Solute free water reabsorption (T^{c}_{H2O}) = [urine flow rate][(Uosm/Posm) -1] in ml/min.

Biochemistry Characteristic	Cohort 2 cases		Cohort 2 controls	
	Hyponatremic TIH cases on thiazides	Normonatremic TIH cases off thiazides	Normonatremic thiazide controls	Normonatremic non-thiazide controls
Sodium %	0.58+/-0.09	0.72+/-0.04	0.65+/-0.03	0.59+/-0.04
Potassium %	11.1+/-1.1	13.8+/-0.6	16.3+/-0.5¶¶¶¶	14.7+/-0.6
Chloride %	0.79+/-0.12	1.10+/-0.08†	0.95+/-0.04	0.88+/-0.05
Calcium %	1.08+/-0.13	1.26+/-0.09	1.01+/-0.06	1.26+/-0.13
Phosphate %	10.8+/-1.2	15.2+/-0.7†	18.0+/-0.8¶¶¶¶¶	19.2+/-1.2*
Magnesium %	3.05+/-0.44	3.49+/-0.17	3.74+/-0.15	3.49+/-0.18
Zinc %	1.23+/-0.14	0.60+/-0.04††††	0.55+/-0.05¶¶¶¶	0.49+/-0.04

Supplementary table S4. Fractional urinary excretion of electrolytes of TIH cases and controls in cohort 2. TIH cases n=109, thiazide controls n=106, non-thiazide controls n=60.† cohort 2 hyponatremic TIH cases on thiazides vs cohort 2 normonatremic TIH cases off thiazides. 1 way ANOVA with Bonferroni correction: † p<0.05, †† p < 0.005 †††$p<10^{-4}$, ††††$p<10^{-5}$.¶ cohort 2 hyponatremic TIH cases on thiazides vs cohort 2 normonatremic thiazide controls: ¶ p<0.05, ¶¶ p < 0.005 ¶¶¶$p<10^{-4}$, ¶¶¶¶$p<10^{-5}$.§ cohort 2 normonatremic non-thiazide controls vs cohort 2 normonatremic TIH cases off thiazides: § p<0.05, §§ p < 0.005 §§§$p<10^{-4}$, §§§§$p<10^{-5}$

Supplementary Figure S1 details serum and urinary uric acid concentrations in cohort 2 patients.

(A) (B)

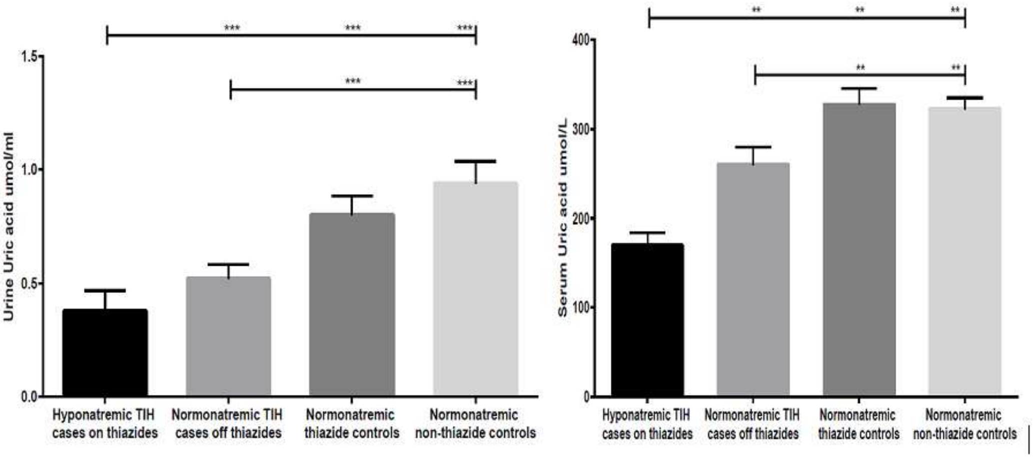

Supplementary figure S1. Serum and urinary uric acid concentration in cohort 2 TIH cases and controls. (A) Urinary uric acid concentration, (B) Serum uric acid concentration. n=20 in each group. 1 way ANOVA with Bonferroni correction. Data represented as mean±SEM. **p < 0.01, ***p < 0.001.

Results of genetic studies

Genome Wide Association Study of 48 cases in first TIH cohort

vs controls from 1958 birth cohort.

Data were available for a total of 1,043,142 SNPs from 48 cohort 1 TIH cases on thiazides and for 1,157,986 SNPs from 2922 controls from the 1958 birth cohort. Exclusions were carried out separately in the case and control datasets. Individuals were excluded if more than 5% of their genotype data was missing. SNPs were excluded if they had more than 5% data missing, had a minor allele frequency (MAF) less than 1%, or if they significantly deviated from Hardy Weinberg Equilibrium (HWE) at the P=0.001 level. The number of individuals/SNPs which did not meet these criteria are summarised in Supplementary Table S5, by case-control status. After exclusions, 818,463 SNPs from 48 cases and 944,677

SNPs from 2905 controls remained, with 506,674 SNPs common to both cases and controls. The case and control genotype data was combined, and a second stage of exclusions were made: 1040 A/T SNPs and 1774 C/G SNPs were removed due to strand assignment issues, along with 1197 SNPs which were identified as having differential missingness in cases and controls (number of missing genotypes significantly differed in cases and controls at the P=0.01 level, Supplementary table 5).

		**Cohort 1 hyponatremic TIH cases on thiazides**	**1958 birth cohort controls**
	Total Individuals, n	48	2922
**Sample QC**	Individuals with >5% missing genotypes, n(%)	0(0%)	17 (0.6%)
	Individuals passing QC	48	2905
	Total SNPs, n	1,043,142	1,157,986
**Stage 1 SNP QC**	SNPs with >5% missing data, n(%)	36,092 (3.5%)	7470 (0.6%)
	SNPs with <1% MAF, n(%)	219,921 (21.1%)	194,387 (16.8%)
	SNPs deviating from HWE at P=0.001, n(%)	771 (0.1%)	18,968 (1.6%)
	SNPs passing stage 1 QC	818,463	944,677
	SNPS common in cases & controls	506,674	
**Stage 2 SNP QC**	A/T and C/G SNPs, n(%)	2814 (0.6%)	
	SNPs with differential missingness (P=0.01), n(%)	1197 (0.2%)	
	SNPs passing stage 2 QC	502,633	

Supplementary table S5. Sample and SNP data Quality Control (QC)

Association Analysis: After quality control filters were applied, 502,663SNPs from 48 cases and 2905 controls, remained for association testing. The analysis gave an inflation factor of λ=1.007 and the resultant QQ plot (Supplementary Figure S2) showed that the distribution of observed P values was fairly close to what was expected. In total, 17 SNPs within 14 regions were identified as showing association with TIH ($P<1.0x10^{-5}$).

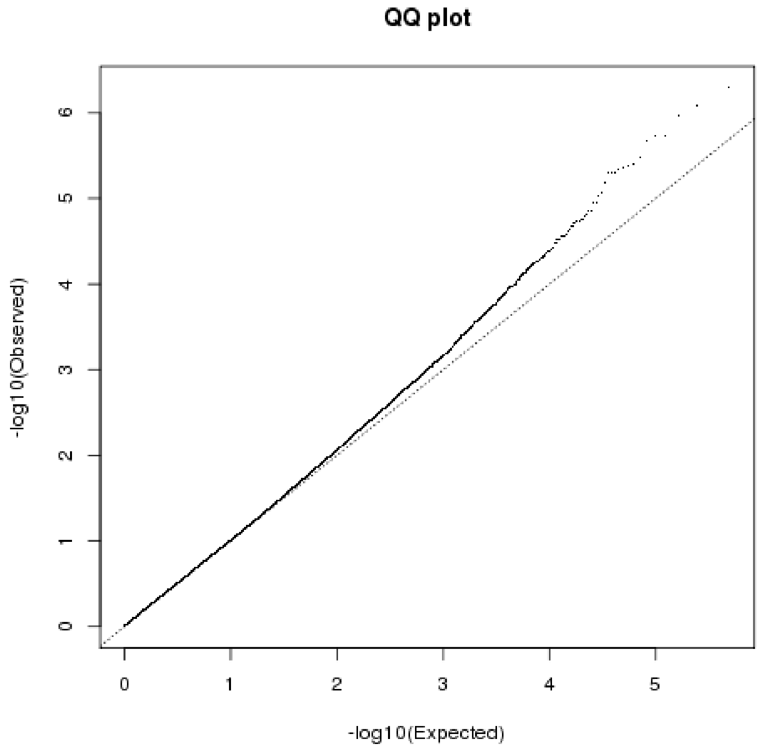

Supplementary figure S2. Expected vs Observed -$\log_{10}$ (P-values)

SNP	Chr	Position	Minor / Major Allele	MAF Cases	MAF Controls	OR (95% CI)	P-Value	Nearest gene
**rs7341607**	8	120667532	A/G	0.073	0.013	6.09 (2.73, 13.58)	5.11×10^{-7}	ENPP2
**rs2196379**	4	175320991	C/A	0.146	0.042	3.89 (2.18, 6.96)	8.06×10^{-7}	FBXO8, CEP44
**rs17175174**	12	47571901	A/G	0.208	0.075	3.26 (1.98, 5.39)	1.06×10^{-6}	CCDC65, RND1
**rs2271171**	4	134295603	G/A	0.073	0.014	5.63 (2.53, 12.53)	1.87×10^{-6}	PCDH10
**rs16970325**	15	38160423	A/G	0.240	0.095	3.00(1.87, 4.84)	2.10×10^{-6}	BCl2BMF
**rs6796067**	3	62864149	G/A	0.073	0.014	5.43 (2.44, 12.07)	3.32×10^{-6}	CADPS
**rs4854769**	3	135153081	C/A	0.385	0.196	2.58 (1.70, 3.90)	3.92×10^{-6}	SLCO2A1
**rs2684796**	15	97300915	A/G	0.250	0.104	2.88 (1.80, 4.60)	4.08×10^{-6}	IGF1R
**rs4747211**	10	73296229	G/A	0.208	0.079	3.06 (1.85, 5.05)	4.43×10^{-6}	PSAP
**rs1490960**	18	61961614	C/A	0.063	0.011	5.88 (2.49, 13.93)	4.88×10^{-6}	CDH7 & CDH19
**rs7760101**	6	166903864	C/A	0.073	0.015	5.28 (2.38, 11.74)	5.00×10^{-6}	RPS6KA2
**rs4951761**	1	209760635	A/G	0.448	0.247	2.47 (1.65, 3.71)	6.62×10^{-6}	RD3 & SLC30A1
**rs2241493**	15	29149644	G/A	0.365	0.186	2.52 (1.65, 3.84)	8.54×10^{-6}	TRPM1
**rs1233755**	20	15501506	A/G	0.063	0.012	5.61 (2.38, 13.27)	9.47×10^{-6}	MACROD2

Supplementary table S6. SNPs associated with TIH in cohort 1, $P<1.0\times10^{-5}$. Only the sentinel SNP within each region is reported.

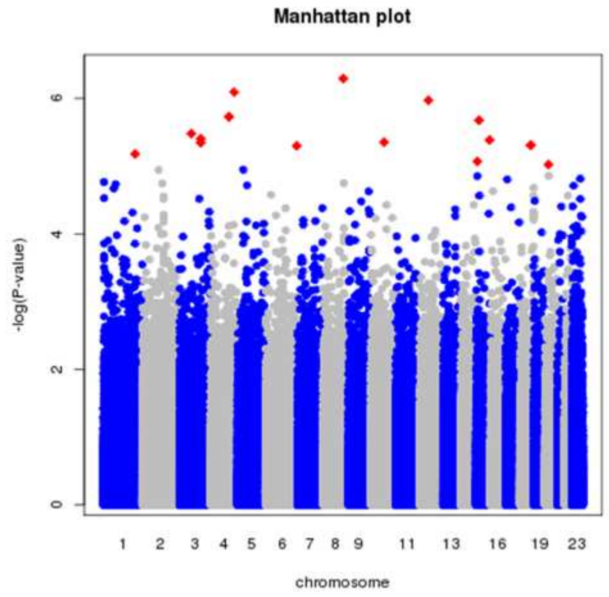

Supplementary figure S3. Manhattan Plot for GWAS of TIH; highlighted SNPs significant at $P=1.0\times10^{-5}$ level

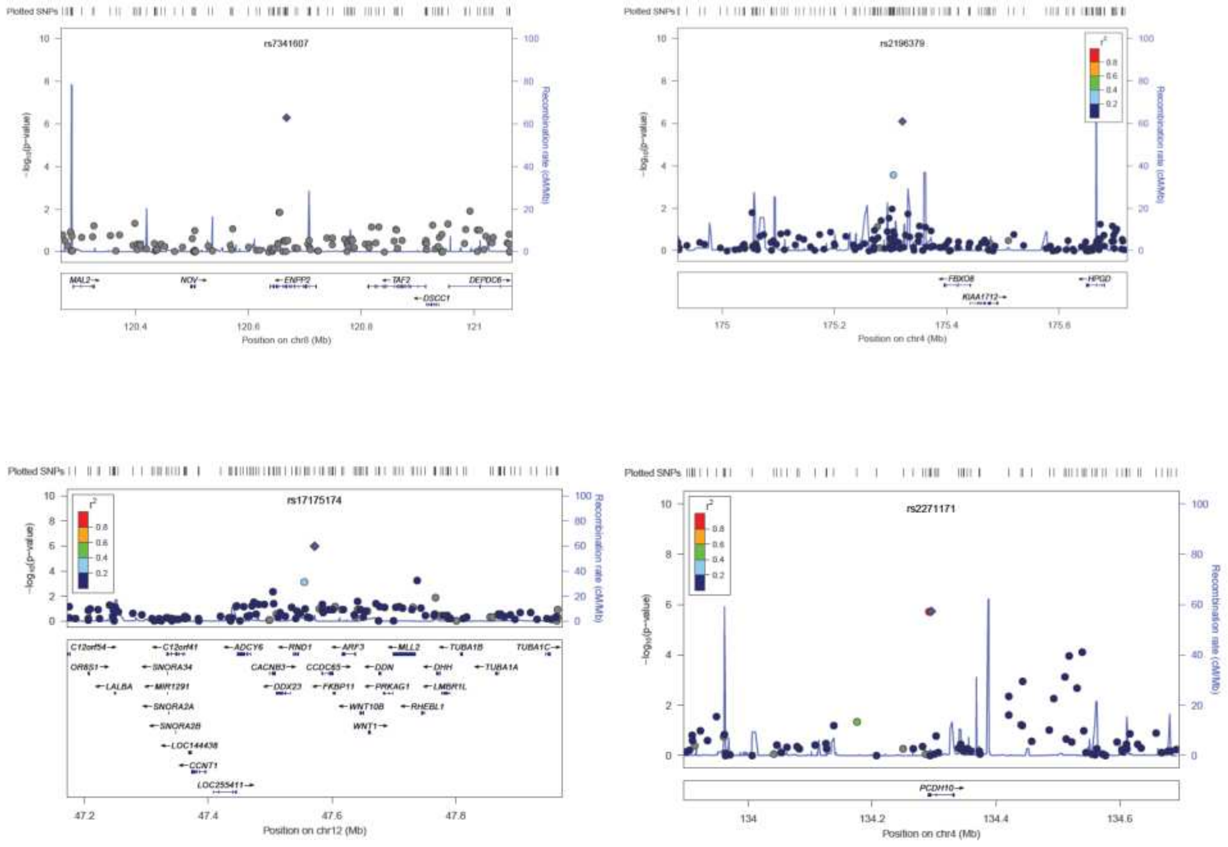

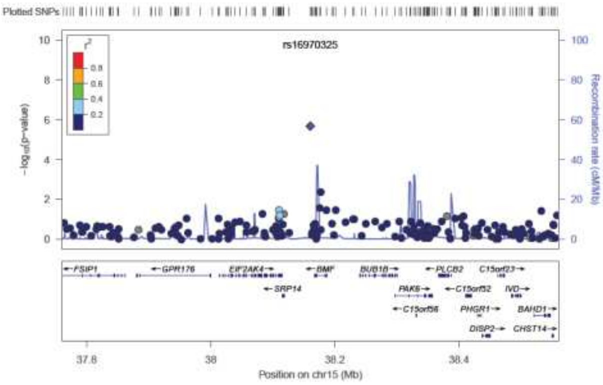
rs16970325
FSIP1
GPR176
EIF2AK4
BMF
BUB1B
PLCB2
C15orf23
SRP14
PAK6
C15orf52
IVD
C15orf56
PHGR1
BAHD1
DISP2
CHST14
Position on chr15 (Mb)
Recombination rate (cM/Mb)

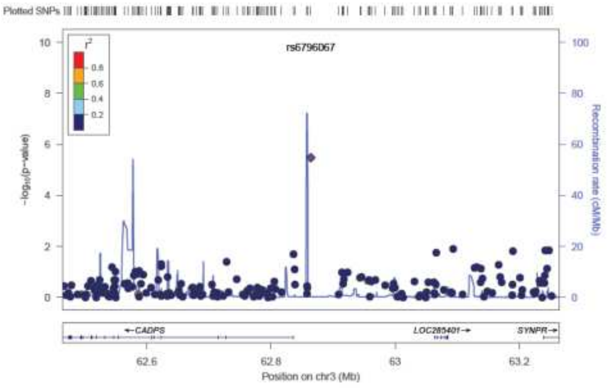
rs6796067
CADPS
LOC285401
SYNPR
Position on chr3 (Mb)
Recombination rate (cM/Mb)

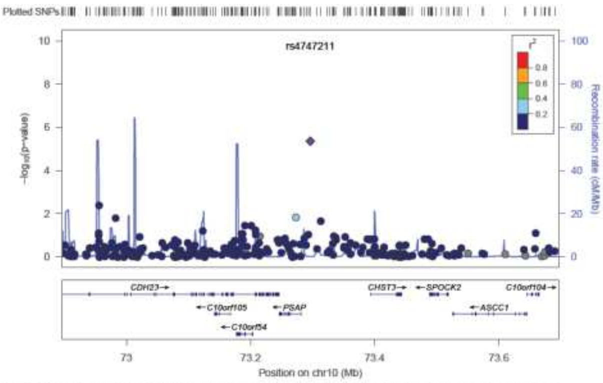
rs4747211
CDH23
CHST3
SPOCK2
C10orf104
C10orf105
PSAP
ASCC1
C10orf54
Position on chr10 (Mb)
Recombination rate (cM/Mb)

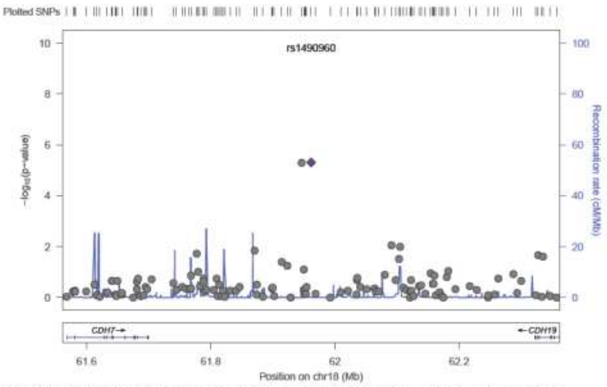
rs1490960
CDH7
CDH19
Position on chr18 (Mb)
Recombination rate (cM/Mb)

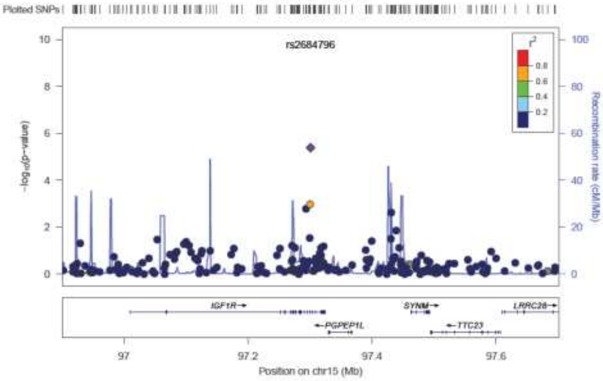
rs2684796
IGF1R
SYNM
LRRC28
PGPEP1L
TTC23
Position on chr15 (Mb)
Recombination rate (cM/Mb)

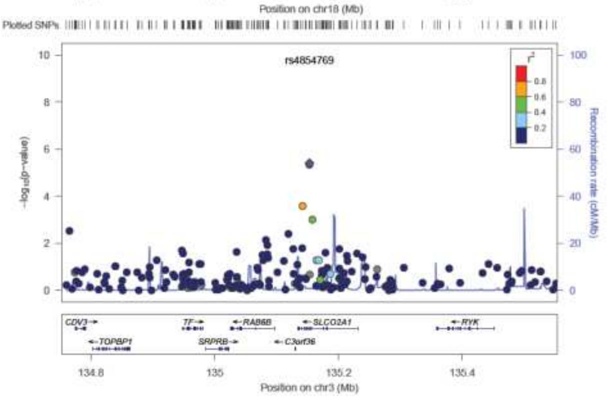
rs4854769
CDV3
TF
RAB6B
SLCO2A1
RYK
TOPBP1
SRPRB
C3orf36
Position on chr3 (Mb)
Recombination rate (cM/Mb)

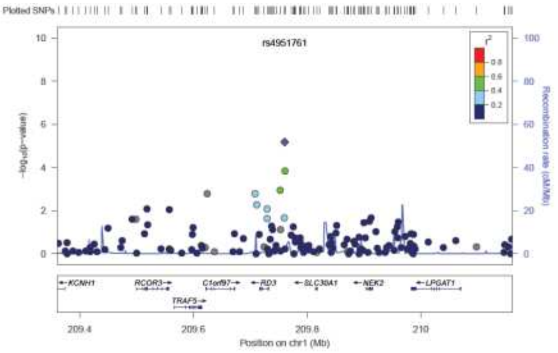
rs4951761
KCNH1
RCOR3
C1orf97
RD3
SLC30A1
NEK2
LPGAT1
TRAF5
Position on chr1 (Mb)
Recombination rate (cM/Mb)

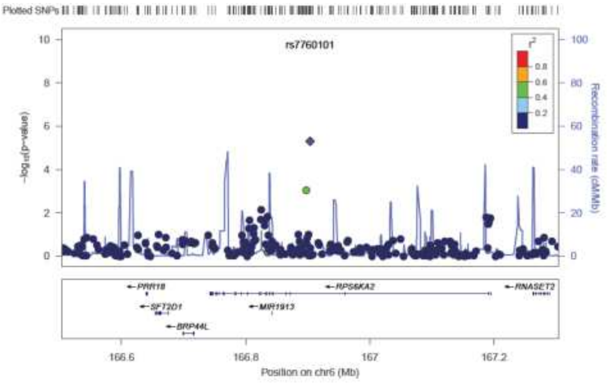
rs7760101
PRR18
RPS6KA2
RNASET2
SFT2D1
MIR1913
BRP44L
Position on chr6 (Mb)
Recombination rate (cM/Mb)

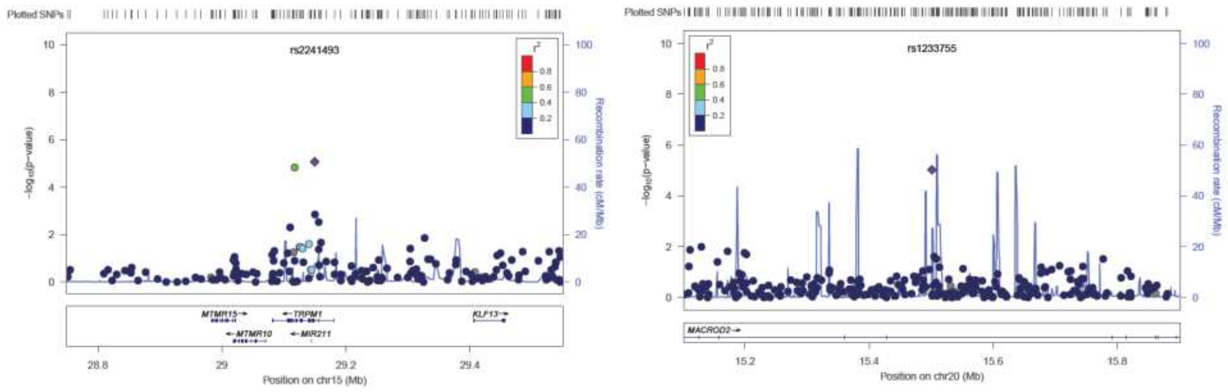

Supplementary figure S4. Region association plots of SNPs associated with cohort 1 hyponatremic TIH cases on thiazides at P=1.0×10^{-5} level detailed in Table S10.

Interrogation of GWAS candidate regions

					Variant details				Number of alleles							Number of individuals			Additive	
POS	REF	ALT	MAF	HWE	GENE	TRANSCRIPT	CONSEQUENCE		MINA	MINU	OBSA	OBSU	REFA	HETA	HOMA	REFU	HETU	HOMU	P	OR
chr3:133666209	C	T	0.34	0.03	SLCO2A1	ENST00000310926	MISSENSE	A396T	34	15	48	53	22	18	8	40	11	2	5.18E-04	3.33
chr15:31362352	C	T	0.73	0.80	TRPM1	ENST00000542188	MISSENSE	S71N	33	22	48	53	5	23	20	3	16	34	0.04	0.50
chr18:64211251	C	T	0.19	1.00	CDH19	ENST00000262150	MISSENSE	V391M	13	26	48	53	35	13	0	31	18	4	0.05	0.48
chr20:15967390	C	T	0.20	0.55	MACROD2	ENST00000310348	MISSENSE	T335M	14	27	48	53	36	10	2	29	21	3	0.08	0.50
chr20:14066276	C	T	0.17	1.00	MACROD2	ENST00000310348	MISSENSE	T58I	20	13	47	53	29	16	2	40	13	0	0.13	1.93
chr15:31369123	A	G	0.74	0.20	TRPM1	ENST00000542188	MISSENSE	M40T	30	23	48	53	1	28	19	3	17	33	0.15	0.61
chr15:31295151	T	G	0.07	1.00	TRPM1	ENST00000542188	MISSENSE	N1268T	4	10	48	53	44	4	0	43	10	0	0.17	0.42
chr15:31294702	G	T	0.04	0.17	TRPM1	ENST00000542188	MISSENSE	P1418T	2	7	48	53	46	2	0	47	5	1	0.17	0.30
chr4:134071945	G	A	0.05	1.00	PCDH10	ENST00000264360	MISSENSE	G217E	3	8	48	53	45	3	0	45	8	0	0.22	0.40
chr2:225362478	C	T	0.12	0.35	CUL3	ENST00000264414	MISSENSE	V567I	10	15	48	53	38	10	0	38	15	0	0.52	0.71
chr12:49308284	A	G	0.33	0.65	CCDC65	ENST00000266984	MISSENSE	H133R	29	37	48	53	25	17	6	22	25	6	0.55	0.81
chr12:49314994	A	G	0.33	0.65	CCDC65	ENST00000266984	MISSENSE	Y408C	29	37	48	53	25	17	6	22	25	6	0.55	0.81
chr4:134071921	G	A	0.08	1.00	PCDH10	ENST00000264360	MISSENSE	G209E	9	7	48	53	39	9	0	46	7	0	0.60	1.46
chr18:63530016	A	G	0.73	0.44	CDH7	ENST00000323011	MISSENSE	N576S	24	30	48	53	4	16	28	5	20	28	0.64	1.18
chr4:134071920	G	A	0.03	1.00	PCDH10	ENST00000264360	MISSENSE	G209R	4	3	48	53	44	4	0	50	3	0	0.71	1.49
chr12:51868968	C	G	0.79	0.01	SLC4A8	ENST00000453097	MISSENSE	T717R	19	24	48	53	0	19	29	0	24	29	0.73	1.19
chr12:49310787	C	A	0.04	1.00	CCDC65	ENST00000266984	MISSENSE	H169N	5	4	48	53	43	5	0	49	4	0	0.74	1.40
chr12:49312540	C	T	0.04	1.00	CCDC65	ENST00000266984	MISSENSE	R294C	5	4	48	53	43	5	0	49	4	0	0.74	1.40
chr15:31294343	A	T	0.05	1.00	TRPM1	ENST00000542188	MISSENSE	H1537Q	4	6	48	53	44	4	0	47	6	0	0.75	0.72
chr15:31294714	C	A	0.05	1.00	TRPM1	ENST00000542188	NONSENSE	E1414*	4	6	48	53	44	4	0	47	6	0	0.75	0.72
chr4:134071923	G	A	0.06	1.00	PCDH10	ENST00000264360	MISSENSE	G210R	7	6	48	53	41	7	0	47	6	0	0.78	1.31
chr3:62459846	A	T	0.45	1.68E-14	CADPS	ENST00000383710	SPLICE DONOR		42	47	46	53	5	40	1	7	45	1	0.89	1.05
chr8:120596022	GA	G	0.97	1.00	ENPP2	ENST00000259486	FRAMESHIFT		3	4	48	53	0	3	45	0	4	49	1.00	1.22
chr15:31334362	C	T	0.03	1.00	TRPM1	ENST00000542188	MISSENSE	V644M	3	4	48	53	45	3	0	49	4	0	1.00	0.82
chr15:31294654	C	T	0.04	1.00	TRPM1	ENST00000542188	MISSENSE	V1434I	4	5	48	53	44	4	0	48	5	0	1.00	0.88
chr6:167271716	T	C	0.83	0.73	RPS6KA2	ENST00000510118	MISSENSE	E32G	16	18	48	53	0	16	32	2	14	37	1.00	1.02
chr1:211751948	A	C	0.57	4.98E-08	SLC30A1	ENST00000367001	MISSENSE	C3G	41	45	48	53	4	33	11	1	43	9	1.00	0.99
chr4:175223209	G	T	0.50	7.03E-30	CEP44	ENST00000426172	MISSENSE	A37S	48	53	48	53	0	48	0	0	53	0	1.00	1.00
chr5:136969744	T	G	0.50	7.03E-30	KLHL3	ENST00000309755	MISSENSE	T478P	48	53	48	53	0	48	0	0	53	0	1.00	1.00
chr15:31323206	A	C	0.50	7.03E-30	TRPM1	ENST00000542188	MISSENSE	V1053G	48	53	48	53	0	48	0	0	53	0	1.00	1.00

Supplementary table S7. One variant within SLCO2A1 is associated with the TIH trait in cohort 1 at a Bonferroni-corrected threshold of P<0.0017 (C>T Chr3:133666209 (rs34550074) encoding p.A396T). In order to fine-map loci identified by GWAS, individual variants in the nearest gene to the peak SNP that were observed at least 7 times in the combined cohort of cohort 1 cases and controls (sufficient observations to achieve a Fisher test nominal p-value of <0.005 if all alleles present in cases, and none in controls) were tested for association with the trait using PlinkSeq.

Abbreviations:REF = reference allele, CONMETA = vcf annotations, ALT = alternate allele, MAF = minor allele frequency, HWE = Hardy-Weinberg equilibrium, MIN = minor allele, OBS = total observed, REF = homozygous reference, HET = heterozygous alternate allele, HOM = homozygous alternate allele; A = affected; U = unaffected , P/OR are p-value & odds ratio under an additive model. PDOM, ORDOM, PREC, ORREC are p-values and odds ratios under dominant and recessive models

Ensembl Gene ID	Ensembl Transcript ID	POS	NVAR	P	I	DESC
ENSG00000174640	ENST00000310926	chr3:133661459..133748570	5	1.9E-03	1.4E-04	0/1(2);1/0(1);1/1(1);34/15(1)
ENSG00000134160	ENST00000542188	chr15:31294159..31369123	18	0.02	1.8E-03	0/0(1);0/1(2);1/0(2);1/2(1);2/0(1);2/7(1);3/4(1)
ENSG00000172264	ENST00000310348	chr20:14066276..15967795	4	0.05	4.1E-03	0/0(1);1/1(1);14/27(1);20/13(1)
ENSG00000071991	ENST00000262150	chr18:64172434..64218391	5	0.09	0.01	0/0(2);1/0(1);1/5(1);13/26(1)
ENSG00000197746	ENST00000394934	chr10:73587913..73588653	2	0.29	0.49	0/0(1);1/0(1)
ENSG00000050438	ENST00000453097	chr12:51856173..51868968	2	0.37	0.04	0/2(1);19/24(1)
ENSG00000164117	ENST00000393674	chr4:175180938..175180938	1	0.38	0.65	1/0(1)
ENSG00000139537	ENST00000266984	chr12:49308284..49315200	6	0.43	0.05	0/2(1);0/3(1);29/37(2);5/4(2)
ENSG00000036257	ENST00000264414	chr2:225346702..225449659	5	0.58	0.27	0/0(2);0/1(1);1/0(1);10/15(1)
ENSG00000146021	ENST00000309755	chr5:136964078..137034083	5	0.67	0.50	0/0(3);0/1(1);48/53(1)
ENSG00000081138	ENST00000323011	chr18:63476982..63530016	2	0.71	0.17	0/1(1);24/30(1)
ENSG00000140443	ENST00000268035	chr15:99250943..99454613	3	0.76	0.04	0/0(1);1/0(1);1/1(1)
ENSG00000138650	ENST00000264360	chr4:134071920..134073706	7	0.83	0.05	0/1(1);12/14(1);3/3(1);3/8(1);4/3(1);7/6(1);9/7(1)
ENSG00000136960	ENST00000259486	chr8:120569823..120638927	4	0.83	0.20	0/0(1);0/1(1);1/3(1);3/4(1)
ENSG00000249141	ENST00000507747	chr6:167271711..167271716	2	0.90	0.03	0/1(1);16/18(1)
ENSG00000170385	ENST00000367001	chr1:211751948..211751948	1	1.00	0.25	41/45(1)
ENSG00000198570	ENST00000367002	chr1:211652382..211654619	2	1.00	0.20	1/2(1);2/3(1)
ENSG00000163618	ENST00000383710	chr3:62459846..62503834	2	1.00	0.13	1/1(1);42/47(1)
ENSG00000171791	ENST00000398117	chr18:60985773..60985773	1	1.00	0.00	0/0(1)
ENSG00000164118	ENST00000426172	chr4:175223209..175229888	4	1.00	0.80	0/0(2);0/1(1);48/53(1)
ENSG00000071242	ENST00000510118	chr6:166831772..167271716	7	1.00	0.06	0/0(2);0/1(3);1/1(1);16/18(1)

Supplementary table S8. Burden testing for genes close to GWAS peaks were assessed for association with TIH, using the C-alpha test to assess whether common and rare variation in combination might be associated with affectation. No significant signals were detected.

Abbreviations: POS=position; NVAR=number of distinct variants; P=nominal p-value; I=an indicator of power, representing the minimum p-value theoretically; obtainable given the observed number of variants & alleles; DESC=a description of the distribution of alleles in affected vs unaffected for each distinct variant site.

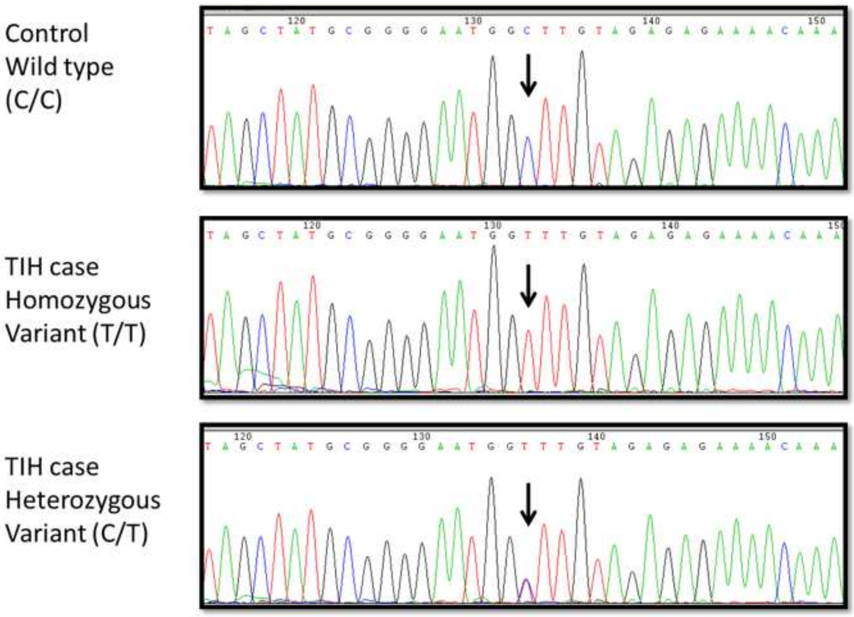

Supplementary figure S5. Sanger sequencing chromatograms of the region containing rs34550074 in hyponatremic TIH cases on thiazides and normonatremic thiazide controls. Wild type (C/C), homozygous variant (T/T) and heterozygous variant (C/T) and shown as indicated by the arrows.

Replication of rs34550074 (p.A396T) in cohort 2 TIH cases and controls

	Cohort 1 (48 cases vs 53 controls)				Cohort 2 (94 cases vs 106 controls)				Cohort 1+2 Combined	
	MAF (MAC)		Association Result		MAF (MAC)		Association Result		Association Result	
	Cases	Controls	OR	P-value	Cases	Controls	OR	P-value	OR	P-value
rs34550074	0.354 (34)	0.142 (15)	3.327	5.18×10^{-4}	0.271 (51)	0.179 (38)	1.702	0.030	2.128	1.70×10^{-4}

Supplementary table 9. Replication of rs34550074 (p.A396T) in cohort 2 TIH cases and controls. Combined Association result estimated using Cochran-Mantel-Haenszel meta-analysis.

Species conservation of SLCO2A1 rs34550074 (A396T)

Human	AALGMLFGGILMKRFVFSLQAIPRIATTIITISMILCVPLF
Mouse	AALGMLFGGILMKRFVFPLQTIPRVAATIMTISIILCAPLF
Rat	AALGMLFGGILMKRFVFPLQTIPRVAATIITISMILCVPLF

Supplementary table 10. Species conservation of SLCO2A1 rs34550074 (A396T). The SNp is highlighted in red. Source: UniProtKB/Swiss-Prot Q92959 http://www.uniprot.org/uniprot/Q92959

Tissue expression of GWAS candidate genes from table S7 including SLCO2A1

Gene	adipose	adrenal	blood	brain	breast	colon	heart	kidney	liver	lung	lymph	ovary	prostate	skeletal_muscle	testes	thyroid
CADPS	0.150724	0.339242	0.078164	66.3673	0.004136	2.42585	7.33205	0.578336	0	0.573661	1.59939	0.889472	2.10418	0.0431645	1.50779	0.058042
CCDC65	0.537446	1.60173	8.91035	4.65269	1.13934	1.1086	0.172254	1.2771	0.256213	3.67274	3.33654	5.49608	0.486168	0.0434132	30.7287	0.987369
CDH19	2.35985	0.823009	0	5.79261	0.047502	22.3591	9.68634	0.209746	1.30585	1.74554	3.42946	0.72678	4.48621	0.223165	1.43356	0.822529
CDH7	0.015701	0.002082	0.011387	1.01757	0.013005	0	0	0	0	0	0	0.024952	0.247223	0	1.09445	0
CEP44	4.73232	3.65926	3.35264	3.23476	4.42077	4.5575	3.16853	5.69168	1.85858	2.20978	5.53978	8.95892	7.20022	2.43424	7.59486	5.69071
ENPP2	147.1997	59.70628	0.316836	211.4306	108.1703	117.427	6.01998	51.29446	11.563	69.79058	55.4035	21.67592	41.0275	6.70912	51.68351	5.91492
FBXO8	8.62634	6.5906	8.65996	5.5443	11.7431	16.4944	12.3274	13.7236	13.8341	10.2798	9.79859	7.88778	18.0382	2.81952	11.6887	12.8725
IGF1R	7.48965	4.93185	6.02384	5.2179	5.90901	8.67359	6.78246	9.19116	0.740085	9.32692	7.04927	8.64473	11.1917	5.1727	8.18034	19.4133
MACROD2	0.618824	2.19421	2.07143	10.3037	1.79405	1.13752	0.59073	3.90292	1.09417	3.22744	0.835012	4.13086	2.80717	0.161879	4.96801	1.35992
PCDH10	0.105645	0.488996	0	9.0669	0.004466	0.615067	0.139646	1.80548	0.0036	0.3593	0.567968	0.960036	3.11455	0.09102	3.26661	0.246871
PSAP	416.347	285.291	1408.64	389.503	460.875	305.745	375.978	327.799	211.455	430.646	281.58	345.625	339.76	347.625	292.189	445.431
RD3	0.005814	0.044135	0	0.002091	0	0.12957	0.025868	0.025605	0.048434	0.028833	0.112073	0.01703	0.0256226	0	0.019827	0
RND1	0.318705	1.3556	0.336718	15.8227	1.94083	1.07612	0.030979	1.00055	8.11731	51.2061	3.70293	0.484561	1.81087	0	1.29479	0.839912
RPS6KA2	29.3585	12.9838	0.760006	15.3732	7.13159	13.9952	21.7918	8.97543	3.53918	15.7524	11.4227	9.75361	11.1919	5.81856	4.28756	15.4989
SLC30A1	13.809	6.94114	17.2959	10.5707	11.2757	7.94114	4.27437	17.4074	39.7654	9.9257	7.31628	17.3002	8.65655	1.72386	13.8156	12.8486
SLCO2A1	9.5118	32.2811	0.067335	0.70289	3.1506	7.09463	5.07971	34.2554	1.38133	47.3888	8.47464	4.20927	14.1765	1.69366	3.40402	15.3912
TRPM1	0.039056	0.026716	0	0.019275	0.018892	0.045238	0	0.017986	0	0	0	0.050933	0	0	1.37085	0

Supplementary table 11. Tissue expression of the GWAS candidate genes from table S7. SLCO2A1 is principally expressed in the kidneys, adrenal glands and lungs. Tissue-specific gene expression data based on Human BodyMap 2.0 - http://www.cureffi.org/2013/07/11/tissue-specific-gene-expression-data-based-on-human-bodymap-2-0/. Units given are Fragments Per Kilobase of exon per Million reads (FPKMs), a measure of gene expression normalized to gene size and RNA-seq library size.

Human kidney immunofluorescence – PGT kidney expression

A - GLOMERULUS (CORTEX)

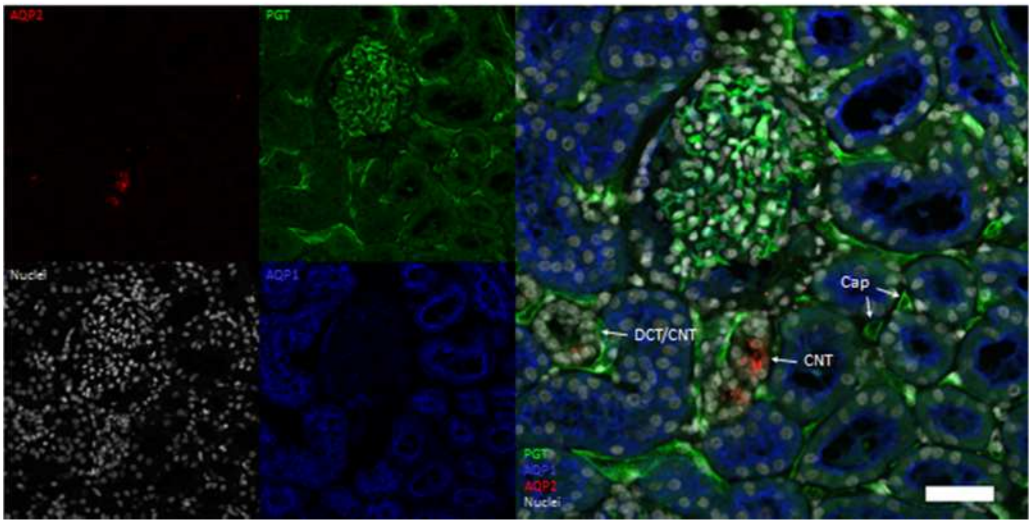

B - CORTEX

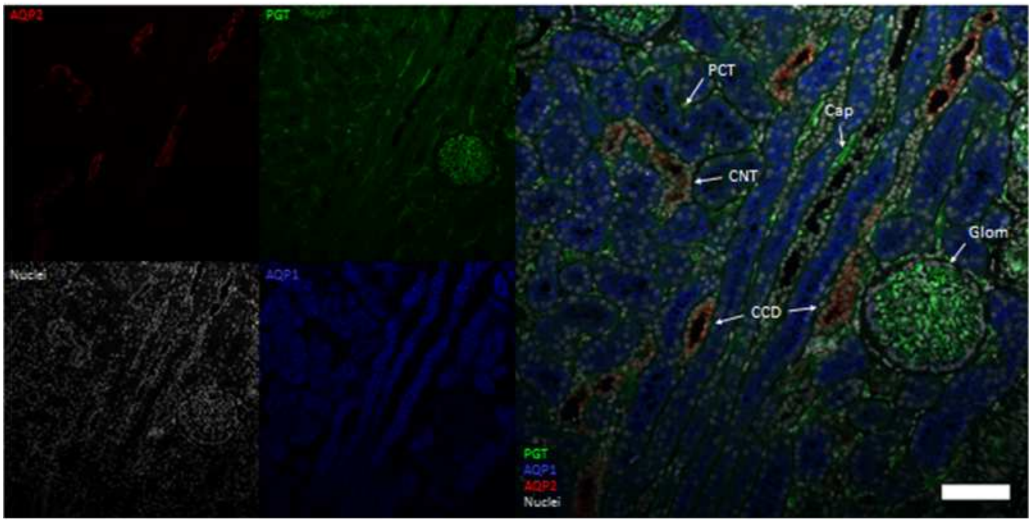

C - CORTEX

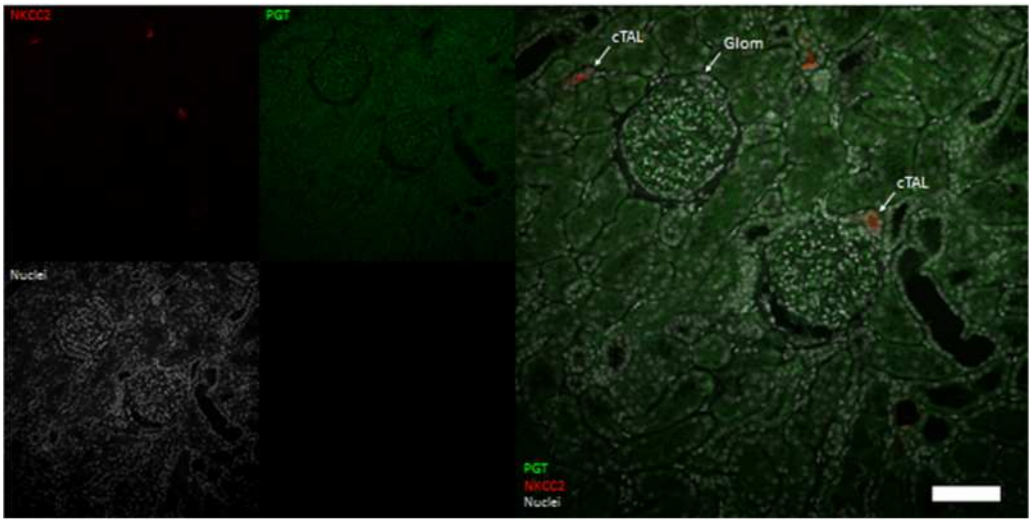

D - CORTEX

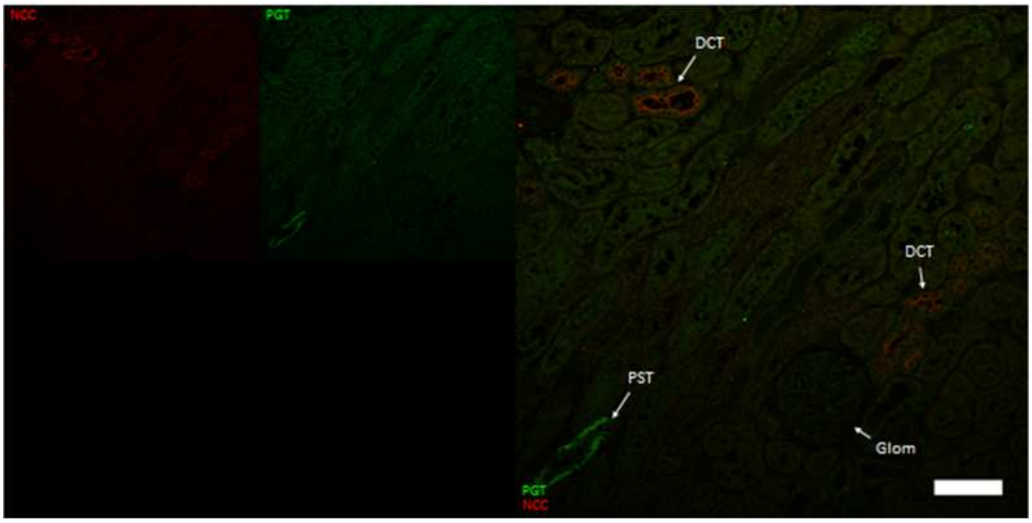

E - CORTEX / OUTER STRIPE OUTER MEDULLA

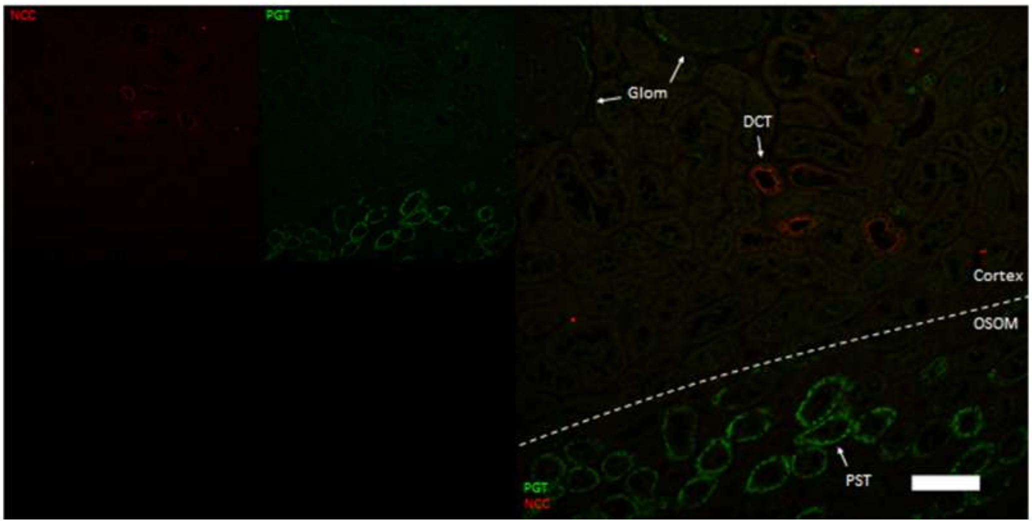

F - OUTER STRIPE OUTER MEDULLA

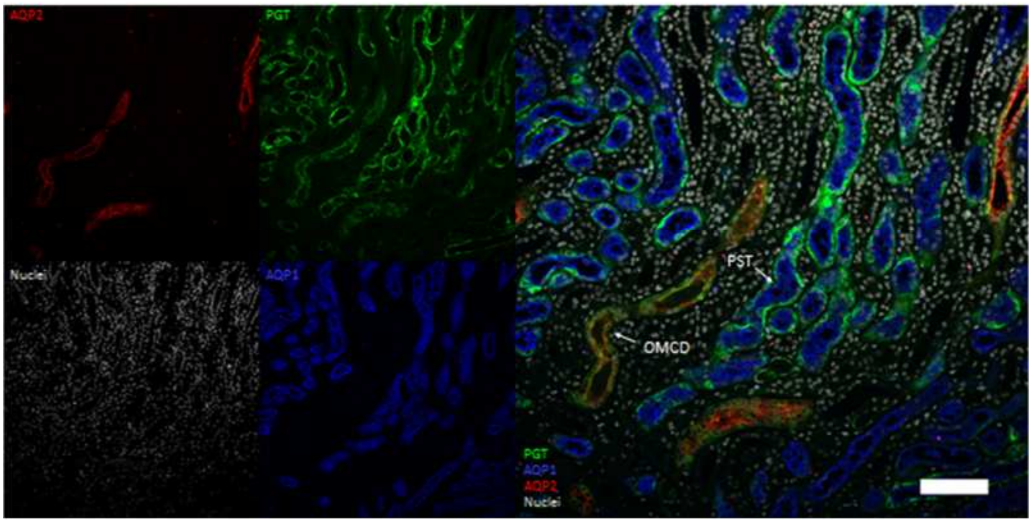

G - OUTER STRIPE OUTER MEDULLA

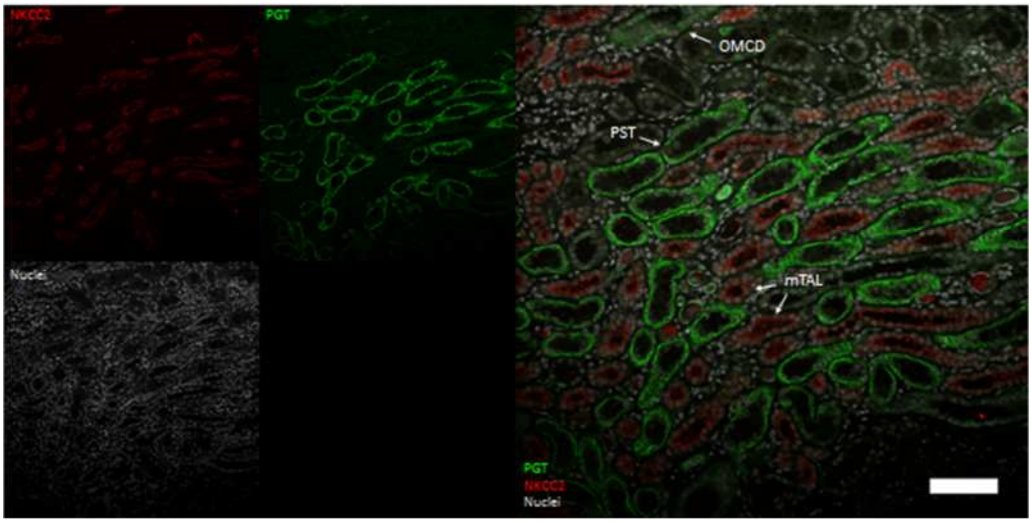

H - INNER STRIPE OUTER MEDULLA

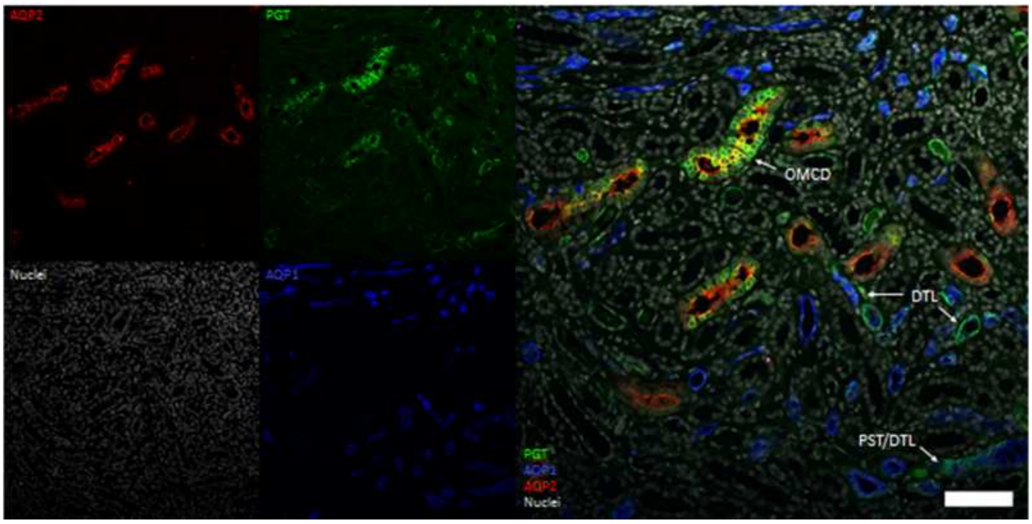

I - INNER STRIPE OUTER MEDULLA

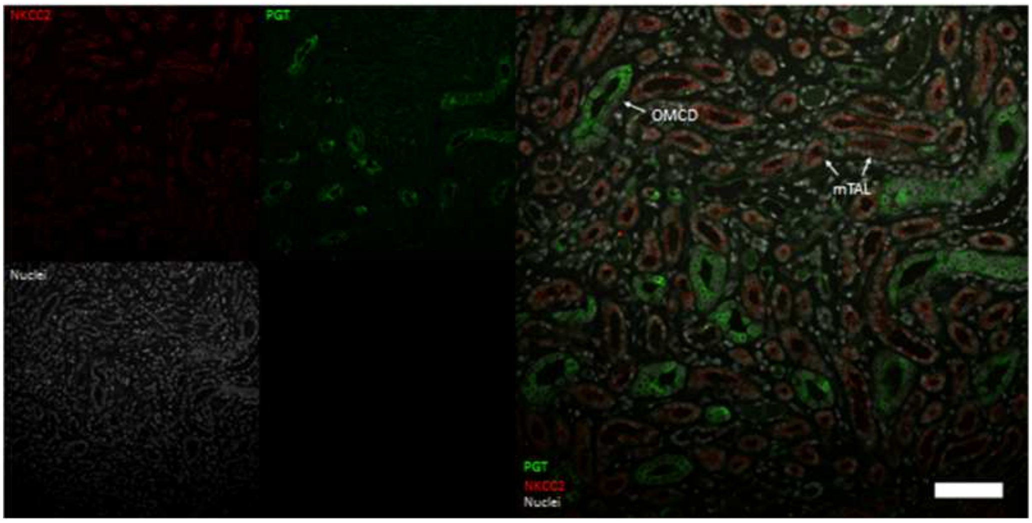

J - INNER STRIPE OUTER MEDULLA / INNER MEDULLA

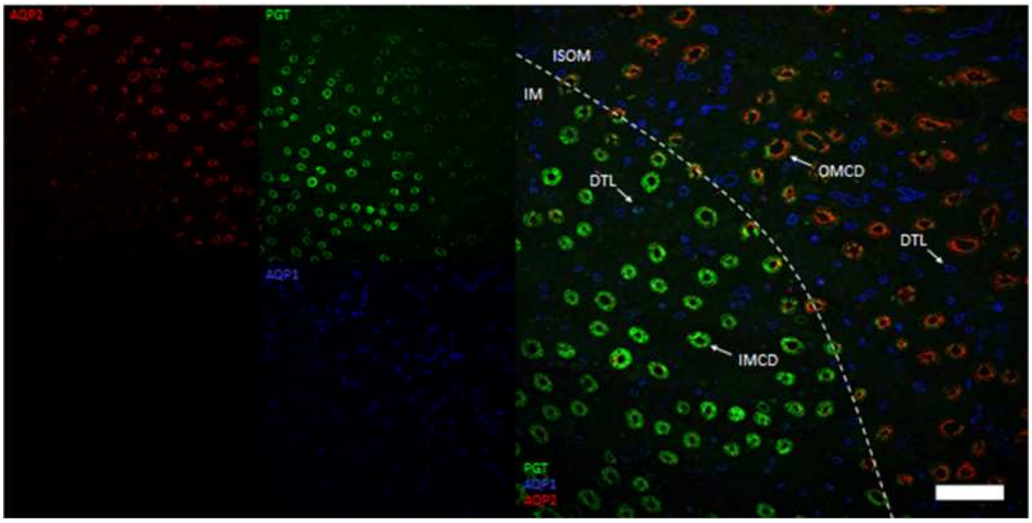

K - INNER STRIPE OUTER MEDULLA / INNER MEDULLA

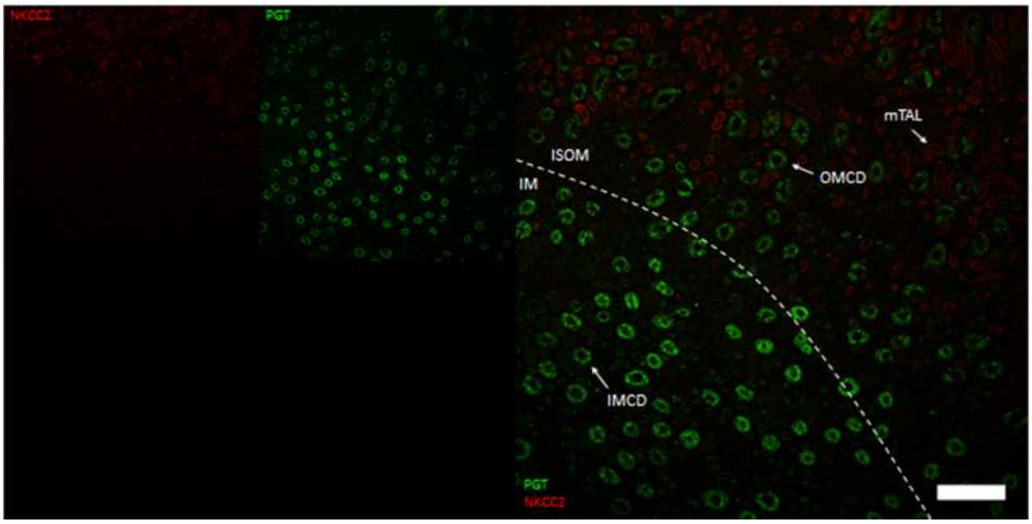

L - INNER MEDULLA

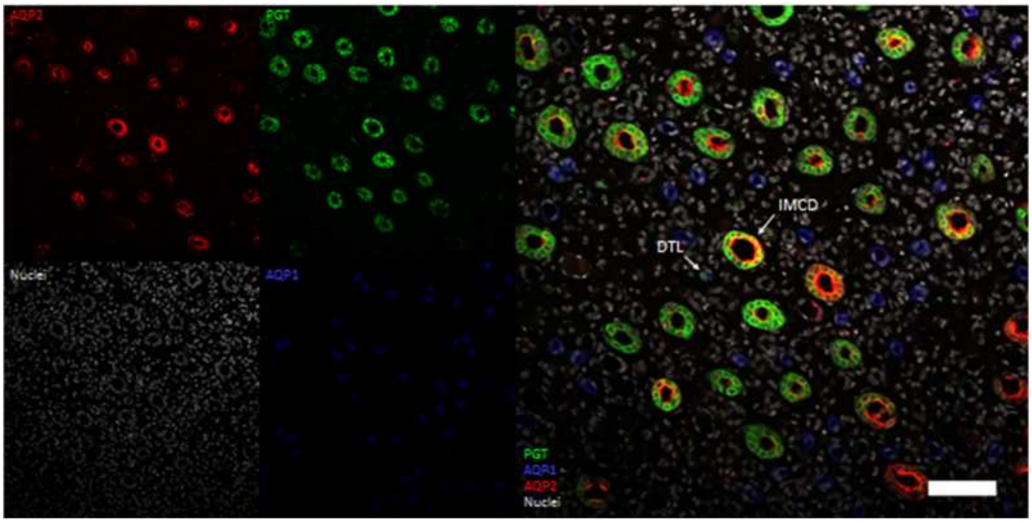

M - THIN LIMBS OF LOOP OF HENLE (INNER MEDULLA)

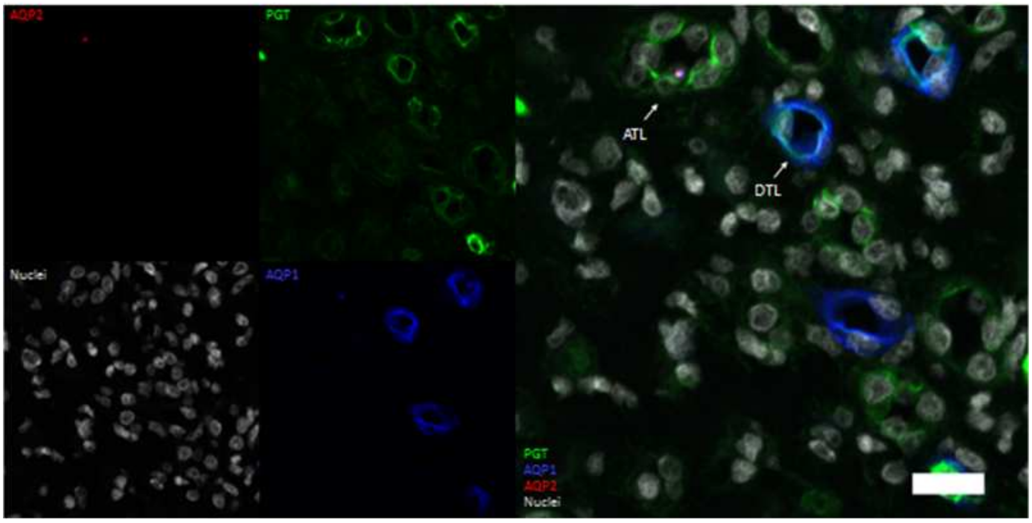

Supplemental figure 6. Representative pseudocolored average intensity z projections of immunofluorescent stained kidney sections showing the distribution of prostaglandin transporters (PGT). PGT was colocalised with the following markers: aquaporin-1 (AQP1) to identify the proximal convoluted tubule (PCT), proximal straight tubule (PST) and descending thin limb loop of Henle (DTL); aquaporin-2 (AQP2) to identify the connecting tubule (CNT) and collecting duct segments - cortical (CCD), outer medullary (OMCD) and inner medullary (IMCD); Na-Cl Co-transporter (NCC) to identify the distal convoluted tubule (DCT); NKCC2 to identify the thick ascending limb loop of Henle segments – cortical (cTAL) and medullary (mTAL). (A – D) Cortex staining showing PGT staining in the glomerulus (Glom) and capillary (Cap), (E) Junction of cortex and outer stripe of the outer medulla

(OSOM), (F – G) OSOM staining, (H – I) Staining of the inner stripe of the outer medulla (ISOM), (J – K) Staining at the interface of the ISOM and inner medulla (IM), (L) IMCD staining in the IM, (M) Higher laser/detector setting digitally zoomed image of the IM AQP1-positive DTL and AQP1-negative ascending thin limb loop of Henle (ATL). Scale Bars: (A) 50 µm; (B – I, L) 100 µm; (J – K) 200 µm; (M) 25 µm. This figure contains images also shown in figure 3.

Urinary PGE_2 and PGE_2 Metabolite (PGE_2M) concentration

Supplementary Figure S10 shows urinary PGE_2 and PGE_2M concentrations in cohort 2 patients.

(A) **(B)**

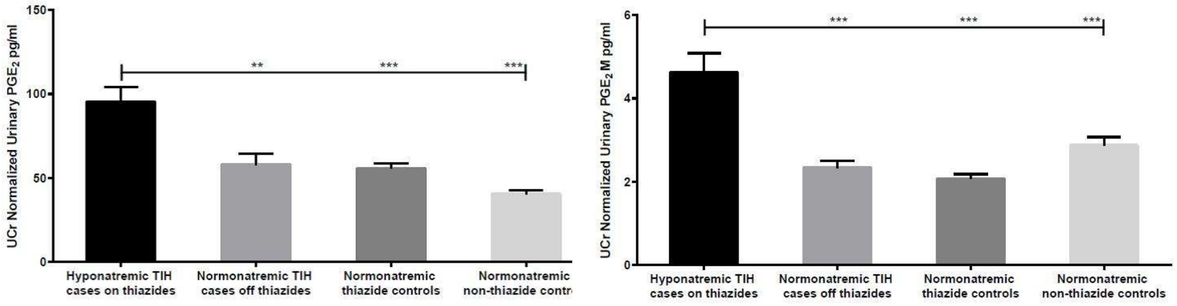

Supplementary figure S7. Urinary prostaglandin concentration in 24 hour urine samples from cohort 2 TIH cases and controls. (A) Prostaglandin E_2 (PGE_2) concentration and (B) PGE_2 Metabolite (PGE_2M) concentration. TIH cases n=47, normonatremic thiazide controls n=96, normonatremic non-thiazide controls n=52. Determined by 1 way ANOVA with Bonferroni correction. Data represented as mean ± SEM. *p < 0.05, **p < 0.01, ***p < 0.001. Ucr – Urinary Creatinine. Ctrl – Control.
